# Supplementary figures and images for: Music score copyright protection based on mixed low-order quaternion Franklin moments
Source: PLoS One. 2025 Aug 25;20(8):e0323447. doi: 10.1371/journal.pone.0323447 (PMC12377592; doi:10.1371/journal.pone.0323447)

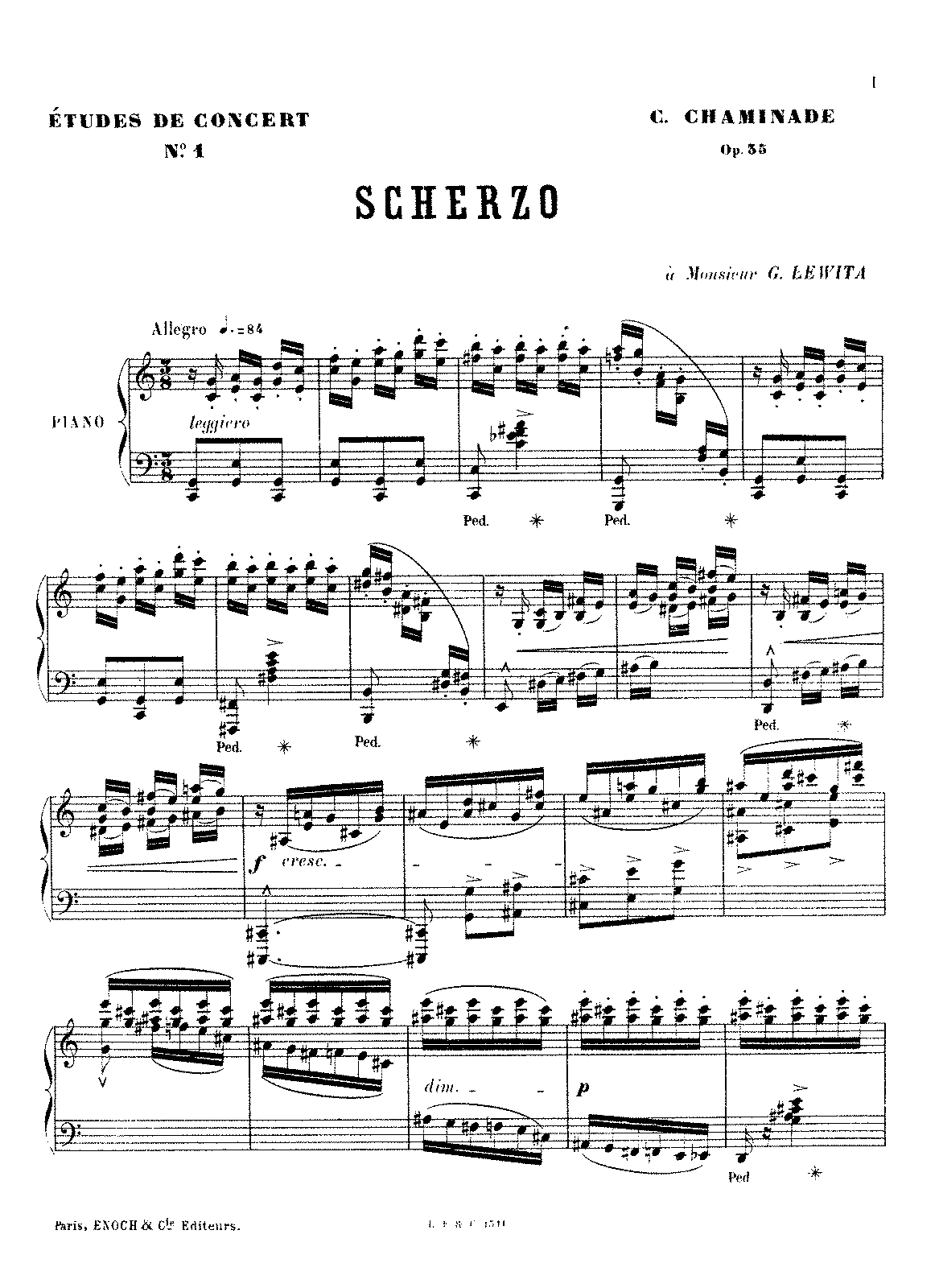

Supplement: S1 Dataset — We obtained the music score images from publicly available music databases, specifically from the IMSLP (International Music Score Library Project). This platform offers a vast collection of music scores that are in the public domain, as well as some modern works with explicit permission. The music score images can be accessed through the following link: https://imslp.org/ The majority of the music score images are sourced from the public domain, and therefore do not have any copyright restrictions. Specifically, the scores selected in our study belong to works in the public domain on the IMSLP platform, which are not protected by current copyright laws (ZIP) [file pone.0323447.s001.zip › Chaminade op35.png]

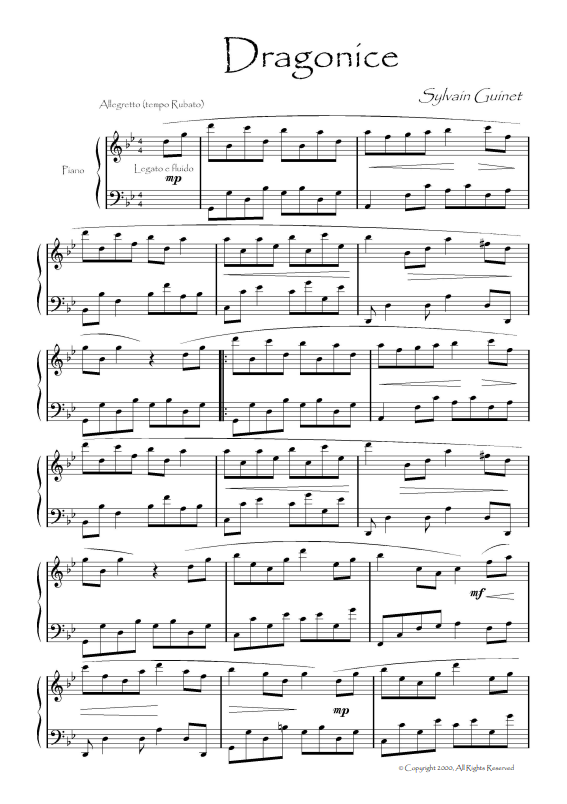

Supplement: S1 Dataset — We obtained the music score images from publicly available music databases, specifically from the IMSLP (International Music Score Library Project). This platform offers a vast collection of music scores that are in the public domain, as well as some modern works with explicit permission. The music score images can be accessed through the following link: https://imslp.org/ The majority of the music score images are sourced from the public domain, and therefore do not have any copyright restrictions. Specifically, the scores selected in our study belong to works in the public domain on the IMSLP platform, which are not protected by current copyright laws (ZIP) [file pone.0323447.s001.zip › Dragonice.png]

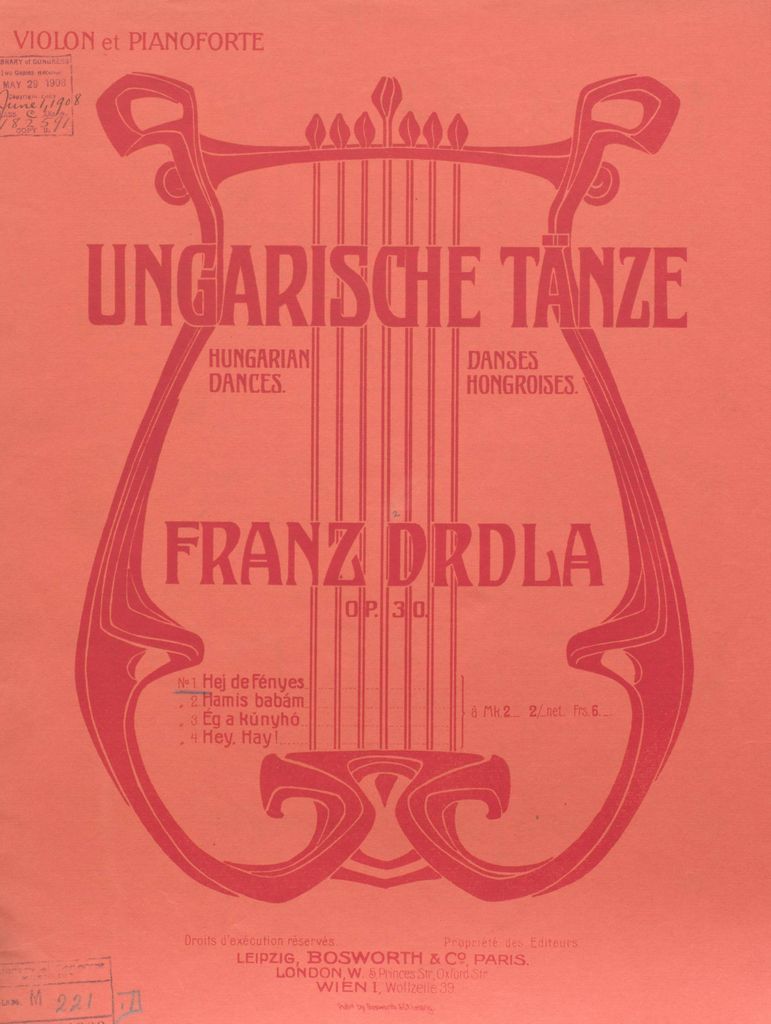

Supplement: S1 Dataset — We obtained the music score images from publicly available music databases, specifically from the IMSLP (International Music Score Library Project). This platform offers a vast collection of music scores that are in the public domain, as well as some modern works with explicit permission. The music score images can be accessed through the following link: https://imslp.org/ The majority of the music score images are sourced from the public domain, and therefore do not have any copyright restrictions. Specifically, the scores selected in our study belong to works in the public domain on the IMSLP platform, which are not protected by current copyright laws (ZIP) [file pone.0323447.s001.zip › Drdla HD red cov.png]

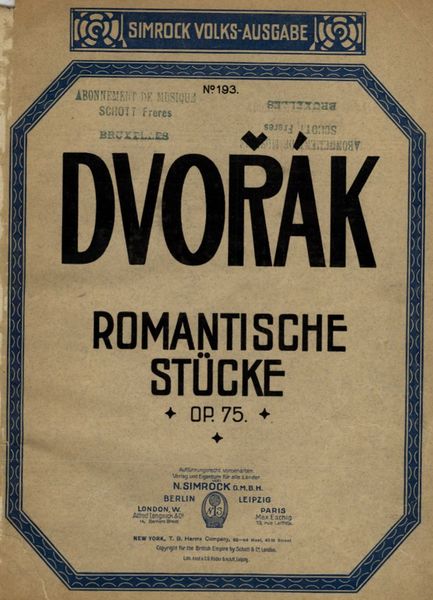

Supplement: S1 Dataset — We obtained the music score images from publicly available music databases, specifically from the IMSLP (International Music Score Library Project). This platform offers a vast collection of music scores that are in the public domain, as well as some modern works with explicit permission. The music score images can be accessed through the following link: https://imslp.org/ The majority of the music score images are sourced from the public domain, and therefore do not have any copyright restrictions. Specifically, the scores selected in our study belong to works in the public domain on the IMSLP platform, which are not protected by current copyright laws (ZIP) [file pone.0323447.s001.zip › Dvo_r_s_sim_cv.png]

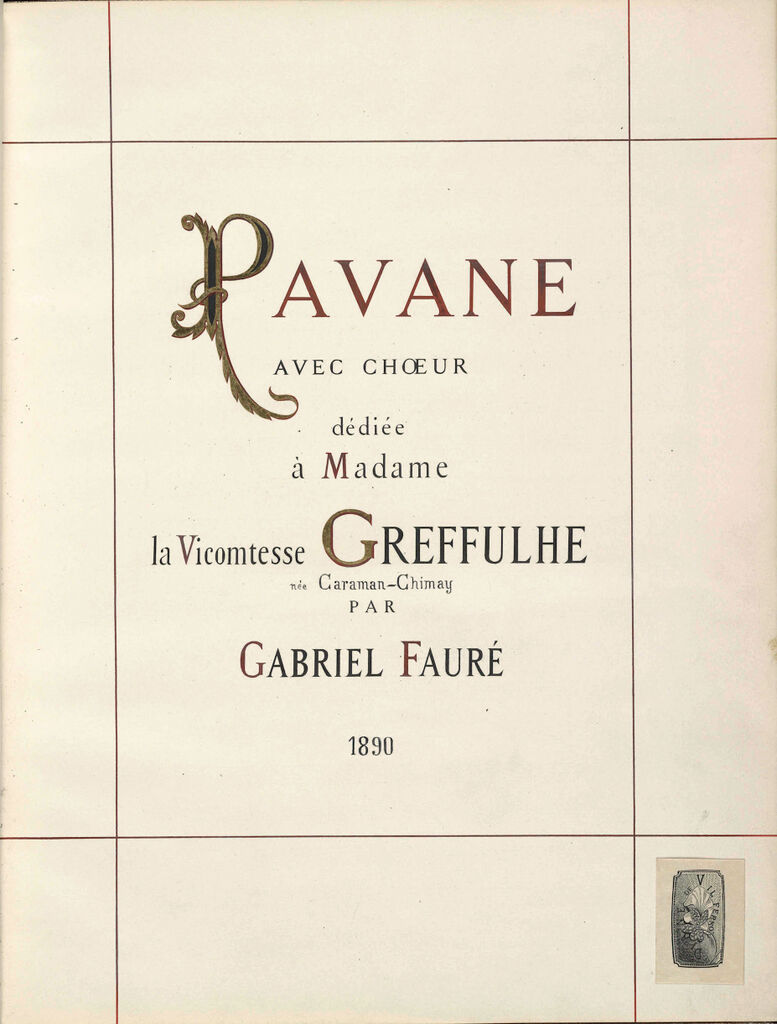

Supplement: S1 Dataset — We obtained the music score images from publicly available music databases, specifically from the IMSLP (International Music Score Library Project). This platform offers a vast collection of music scores that are in the public domain, as well as some modern works with explicit permission. The music score images can be accessed through the following link: https://imslp.org/ The majority of the music score images are sourced from the public domain, and therefore do not have any copyright restrictions. Specifically, the scores selected in our study belong to works in the public domain on the IMSLP platform, which are not protected by current copyright laws (ZIP) [file pone.0323447.s001.zip › Faure-N100mssTP.png]

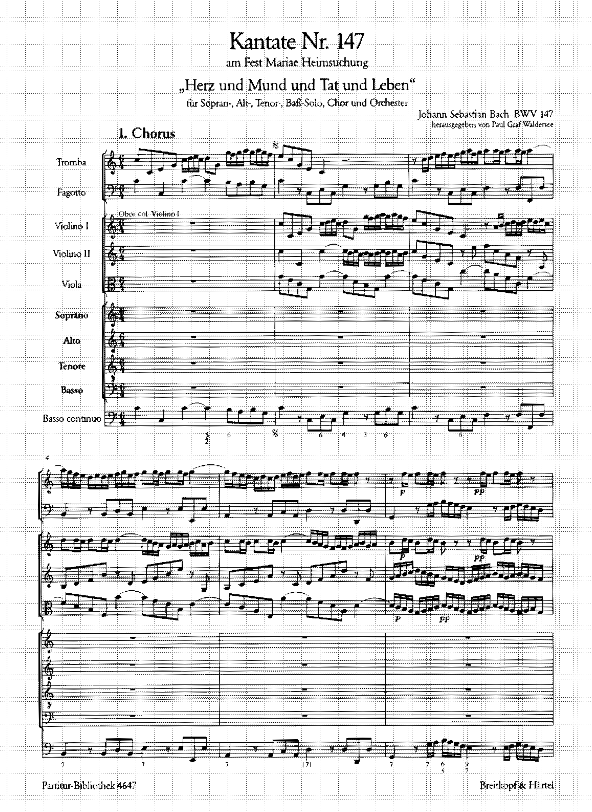

Supplement: S1 Dataset — We obtained the music score images from publicly available music databases, specifically from the IMSLP (International Music Score Library Project). This platform offers a vast collection of music scores that are in the public domain, as well as some modern works with explicit permission. The music score images can be accessed through the following link: https://imslp.org/ The majority of the music score images are sourced from the public domain, and therefore do not have any copyright restrictions. Specifically, the scores selected in our study belong to works in the public domain on the IMSLP platform, which are not protected by current copyright laws (ZIP) [file pone.0323447.s001.zip › Herz_und_Mund.png]

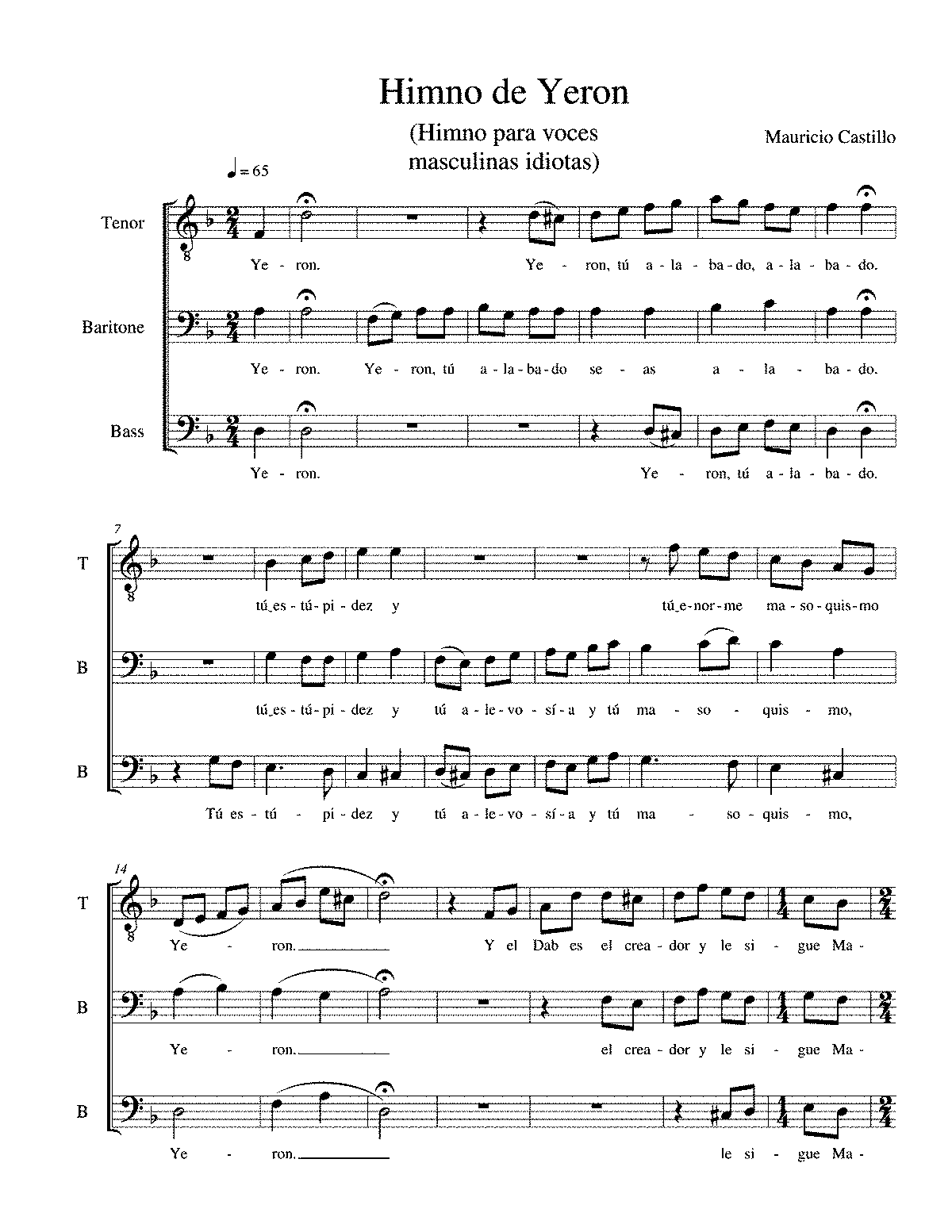

Supplement: S1 Dataset — We obtained the music score images from publicly available music databases, specifically from the IMSLP (International Music Score Library Project). This platform offers a vast collection of music scores that are in the public domain, as well as some modern works with explicit permission. The music score images can be accessed through the following link: https://imslp.org/ The majority of the music score images are sourced from the public domain, and therefore do not have any copyright restrictions. Specifically, the scores selected in our study belong to works in the public domain on the IMSLP platform, which are not protected by current copyright laws (ZIP) [file pone.0323447.s001.zip › Himno de Yeron.png]

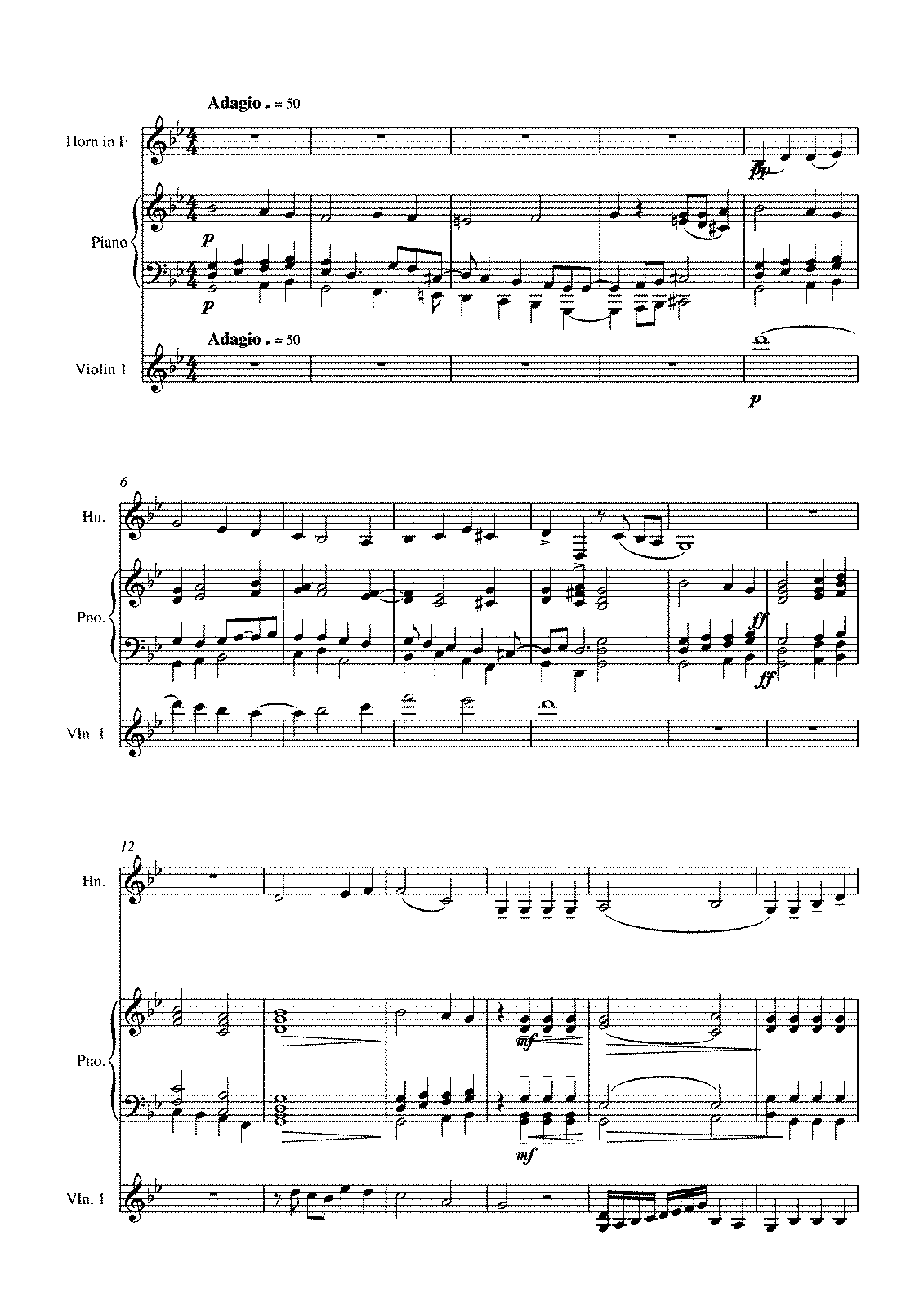

Supplement: S1 Dataset — We obtained the music score images from publicly available music databases, specifically from the IMSLP (International Music Score Library Project). This platform offers a vast collection of music scores that are in the public domain, as well as some modern works with explicit permission. The music score images can be accessed through the following link: https://imslp.org/ The majority of the music score images are sourced from the public domain, and therefore do not have any copyright restrictions. Specifically, the scores selected in our study belong to works in the public domain on the IMSLP platform, which are not protected by current copyright laws (ZIP) [file pone.0323447.s001.zip › Horn Trio.png]

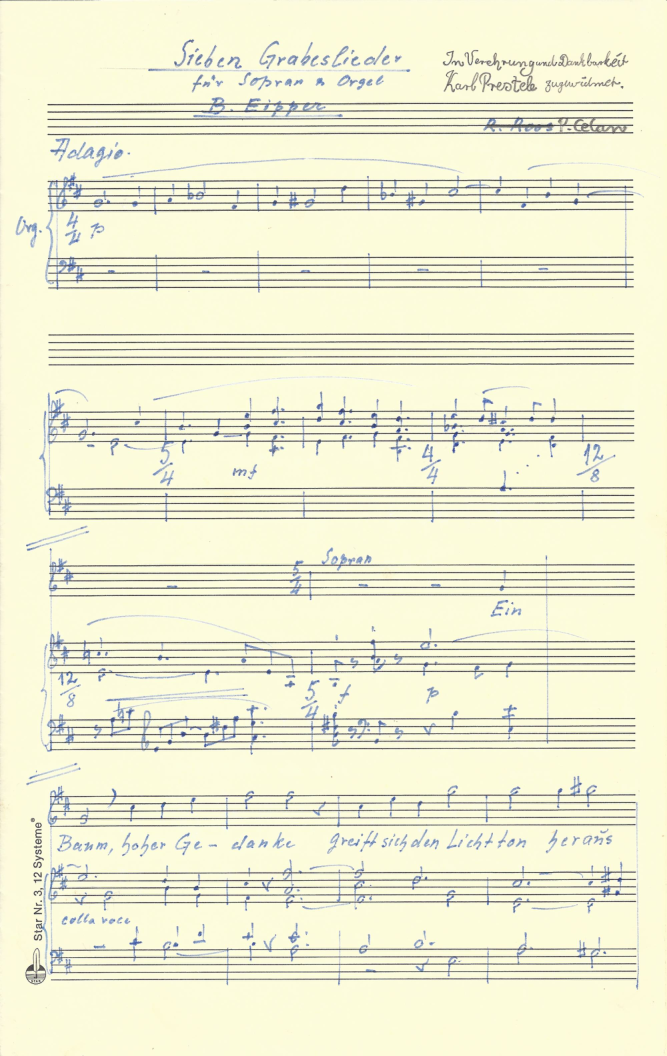

Supplement: S1 Dataset — We obtained the music score images from publicly available music databases, specifically from the IMSLP (International Music Score Library Project). This platform offers a vast collection of music scores that are in the public domain, as well as some modern works with explicit permission. The music score images can be accessed through the following link: https://imslp.org/ The majority of the music score images are sourced from the public domain, and therefore do not have any copyright restrictions. Specifically, the scores selected in our study belong to works in the public domain on the IMSLP platform, which are not protected by current copyright laws (ZIP) [file pone.0323447.s001.zip › IMSLP66023.png]

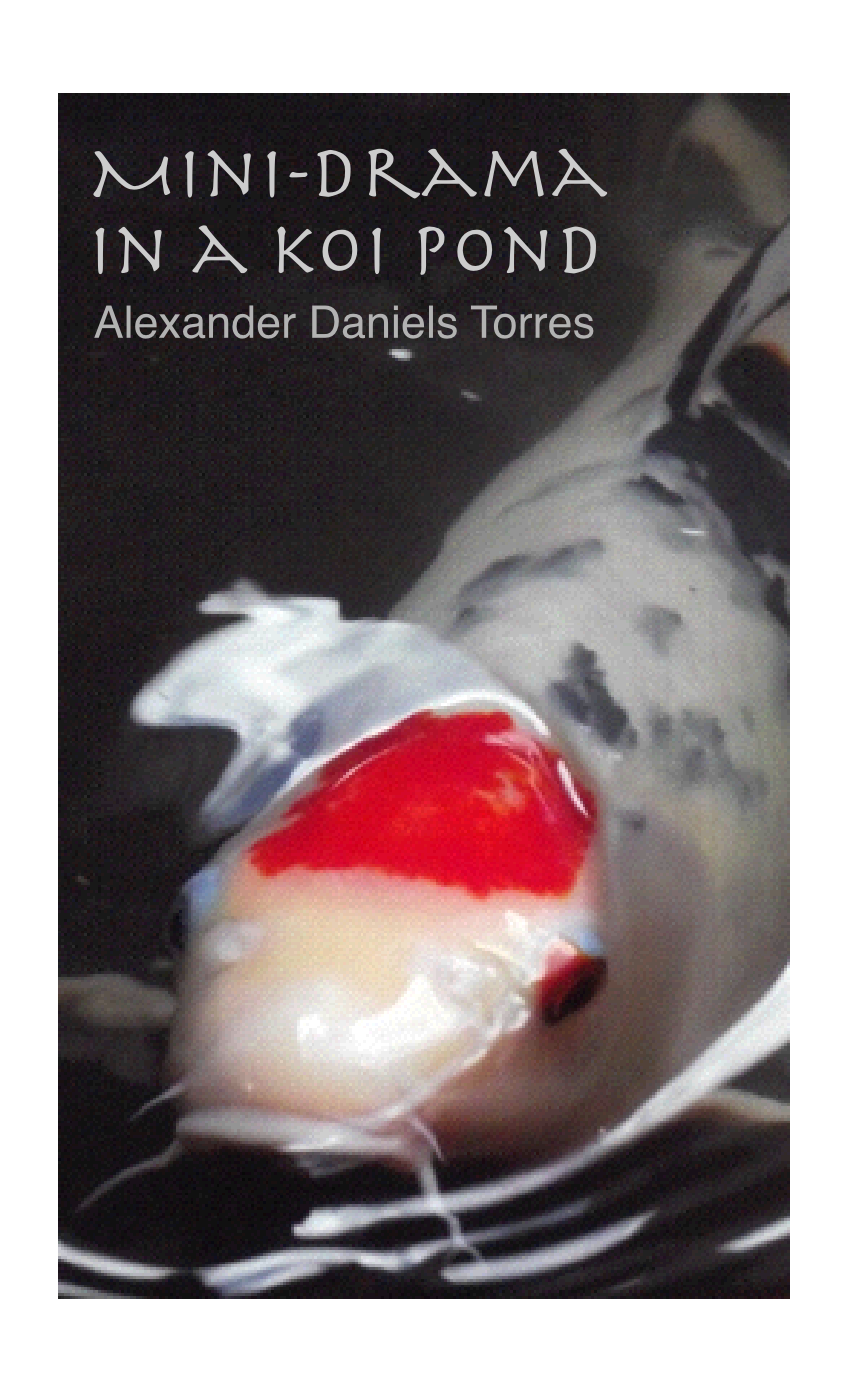

Supplement: S1 Dataset — We obtained the music score images from publicly available music databases, specifically from the IMSLP (International Music Score Library Project). This platform offers a vast collection of music scores that are in the public domain, as well as some modern works with explicit permission. The music score images can be accessed through the following link: https://imslp.org/ The majority of the music score images are sourced from the public domain, and therefore do not have any copyright restrictions. Specifically, the scores selected in our study belong to works in the public domain on the IMSLP platform, which are not protected by current copyright laws (ZIP) [file pone.0323447.s001.zip › IMSLP94026.png]

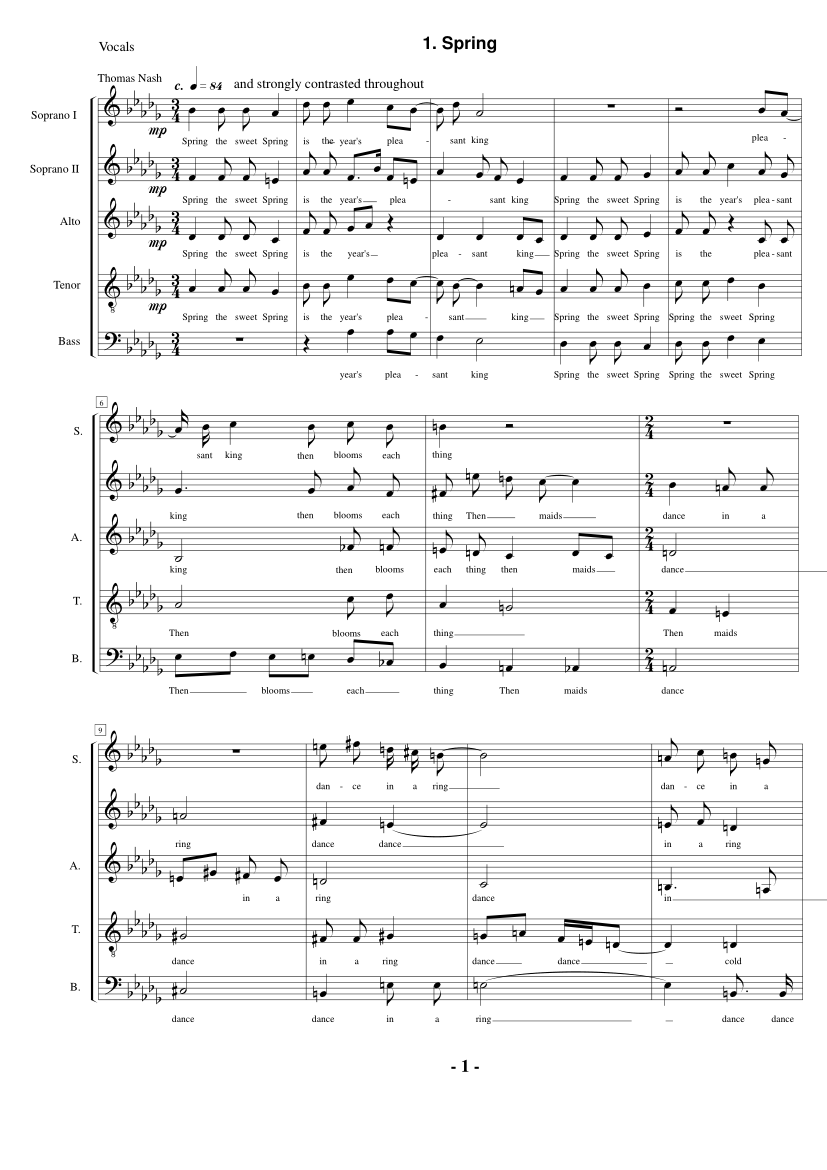

Supplement: S1 Dataset — We obtained the music score images from publicly available music databases, specifically from the IMSLP (International Music Score Library Project). This platform offers a vast collection of music scores that are in the public domain, as well as some modern works with explicit permission. The music score images can be accessed through the following link: https://imslp.org/ The majority of the music score images are sourced from the public domain, and therefore do not have any copyright restrictions. Specifically, the scores selected in our study belong to works in the public domain on the IMSLP platform, which are not protected by current copyright laws (ZIP) [file pone.0323447.s001.zip › IMSLP161551.png]

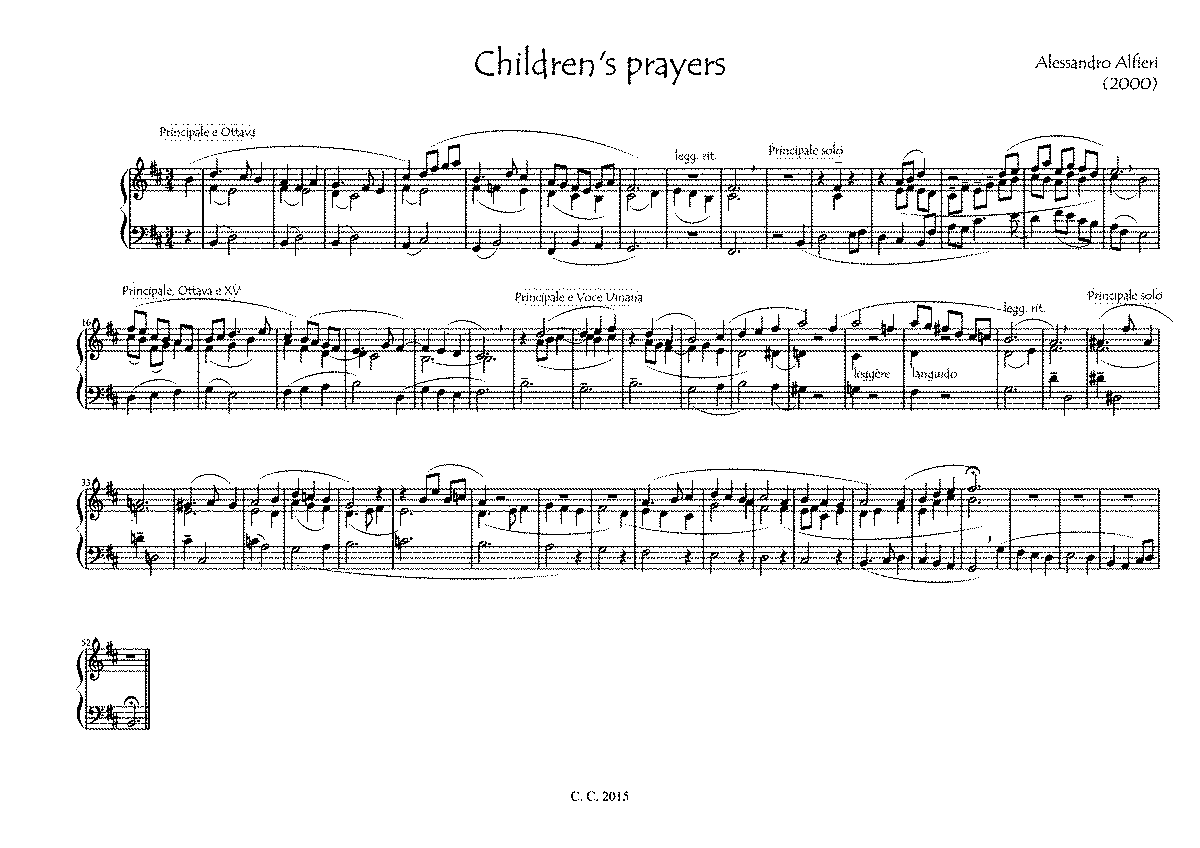

Supplement: S1 Dataset — We obtained the music score images from publicly available music databases, specifically from the IMSLP (International Music Score Library Project). This platform offers a vast collection of music scores that are in the public domain, as well as some modern works with explicit permission. The music score images can be accessed through the following link: https://imslp.org/ The majority of the music score images are sourced from the public domain, and therefore do not have any copyright restrictions. Specifically, the scores selected in our study belong to works in the public domain on the IMSLP platform, which are not protected by current copyright laws (ZIP) [file pone.0323447.s001.zip › IMSLP387735.png]

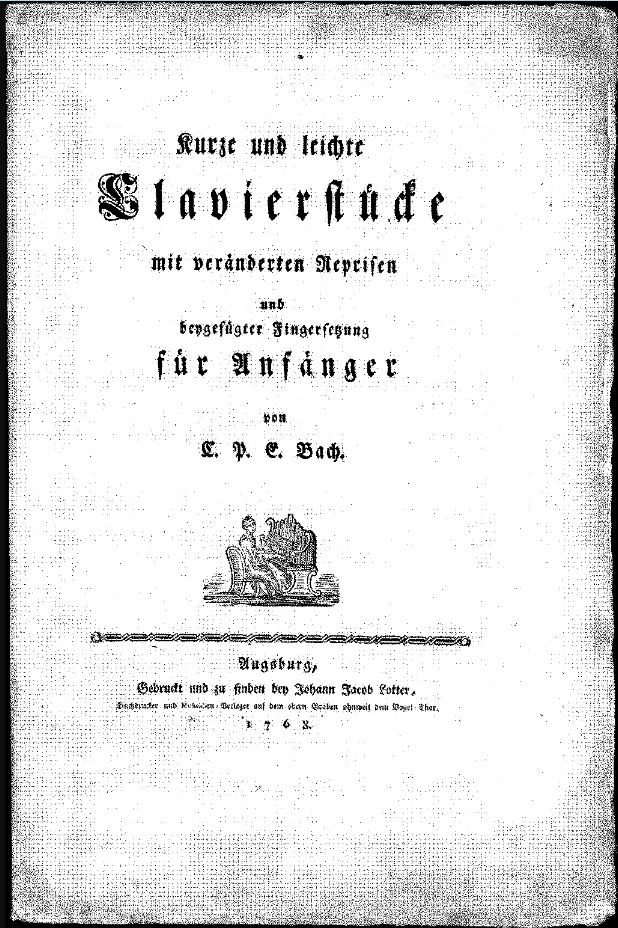

Supplement: S1 Dataset — We obtained the music score images from publicly available music databases, specifically from the IMSLP (International Music Score Library Project). This platform offers a vast collection of music scores that are in the public domain, as well as some modern works with explicit permission. The music score images can be accessed through the following link: https://imslp.org/ The majority of the music score images are sourced from the public domain, and therefore do not have any copyright restrictions. Specifically, the scores selected in our study belong to works in the public domain on the IMSLP platform, which are not protected by current copyright laws (ZIP) [file pone.0323447.s001.zip › Klavierstücke.png]

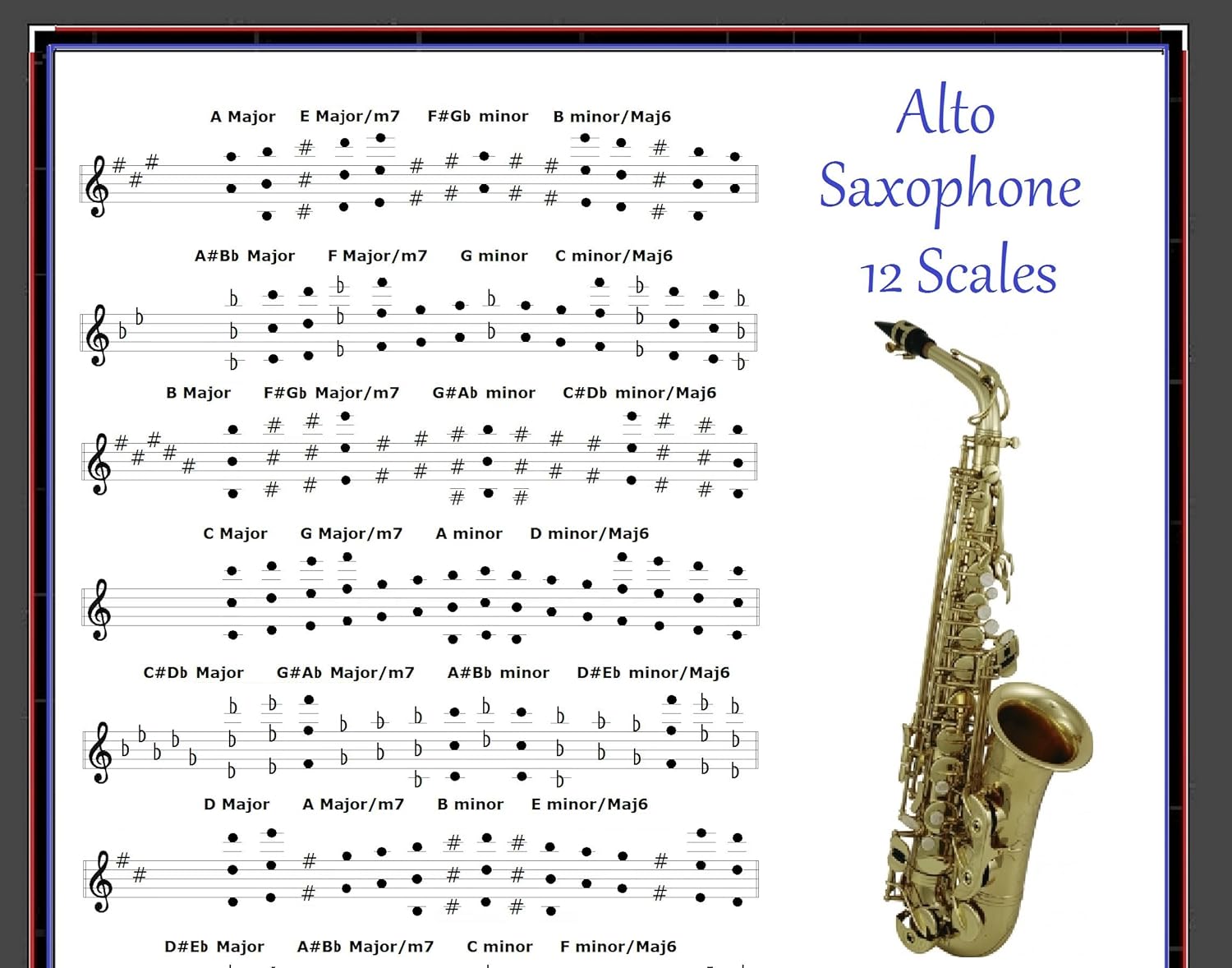

Supplement: S1 Dataset — We obtained the music score images from publicly available music databases, specifically from the IMSLP (International Music Score Library Project). This platform offers a vast collection of music scores that are in the public domain, as well as some modern works with explicit permission. The music score images can be accessed through the following link: https://imslp.org/ The majority of the music score images are sourced from the public domain, and therefore do not have any copyright restrictions. Specifically, the scores selected in our study belong to works in the public domain on the IMSLP platform, which are not protected by current copyright laws (ZIP) [file pone.0323447.s001.zip › music_chart.png]

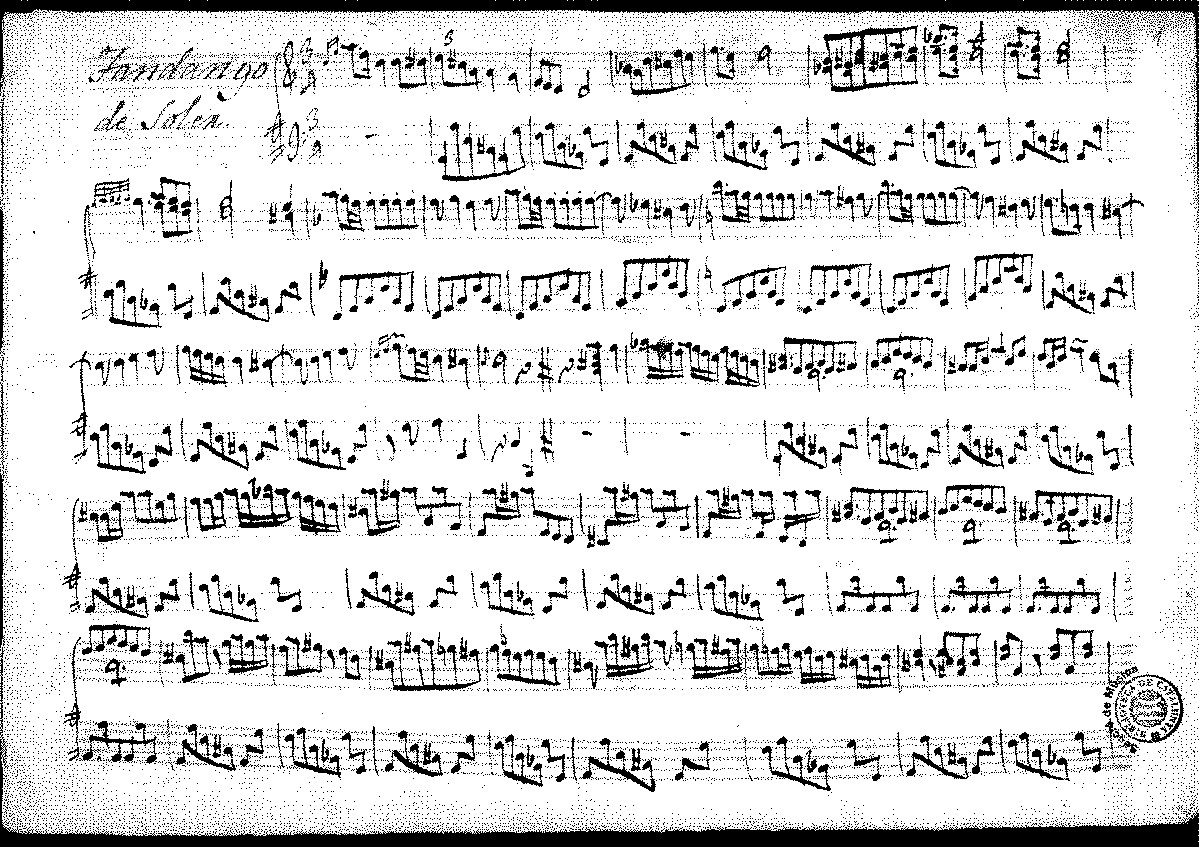

Supplement: S1 Dataset — We obtained the music score images from publicly available music databases, specifically from the IMSLP (International Music Score Library Project). This platform offers a vast collection of music scores that are in the public domain, as well as some modern works with explicit permission. The music score images can be accessed through the following link: https://imslp.org/ The majority of the music score images are sourced from the public domain, and therefore do not have any copyright restrictions. Specifically, the scores selected in our study belong to works in the public domain on the IMSLP platform, which are not protected by current copyright laws (ZIP) [file pone.0323447.s001.zip › PMLP11877.png]

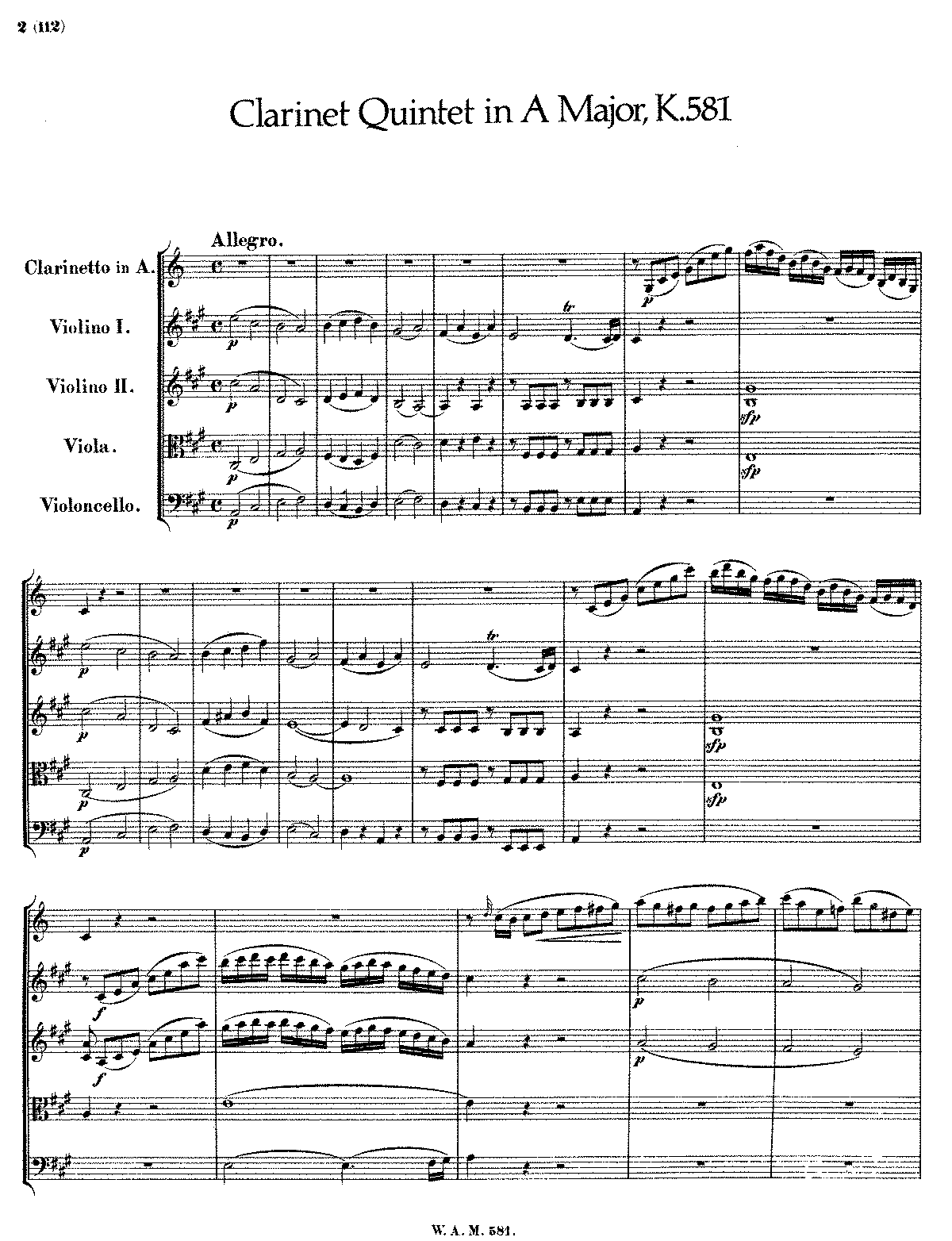

Supplement: S1 Dataset — We obtained the music score images from publicly available music databases, specifically from the IMSLP (International Music Score Library Project). This platform offers a vast collection of music scores that are in the public domain, as well as some modern works with explicit permission. The music score images can be accessed through the following link: https://imslp.org/ The majority of the music score images are sourced from the public domain, and therefore do not have any copyright restrictions. Specifically, the scores selected in our study belong to works in the public domain on the IMSLP platform, which are not protected by current copyright laws (ZIP) [file pone.0323447.s001.zip › PMLP41876.png]

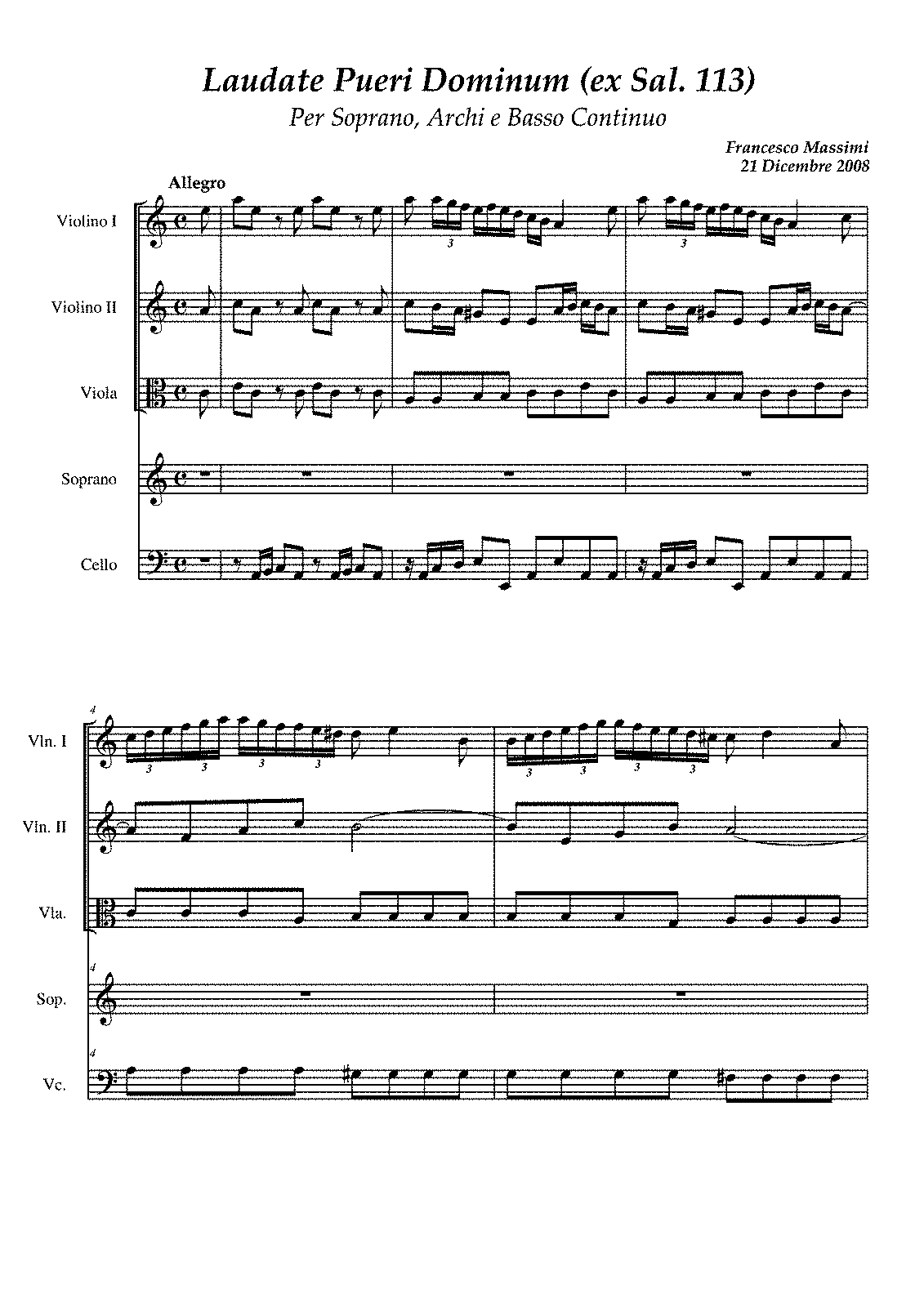

Supplement: S1 Dataset — We obtained the music score images from publicly available music databases, specifically from the IMSLP (International Music Score Library Project). This platform offers a vast collection of music scores that are in the public domain, as well as some modern works with explicit permission. The music score images can be accessed through the following link: https://imslp.org/ The majority of the music score images are sourced from the public domain, and therefore do not have any copyright restrictions. Specifically, the scores selected in our study belong to works in the public domain on the IMSLP platform, which are not protected by current copyright laws (ZIP) [file pone.0323447.s001.zip › PMLP58108.png]

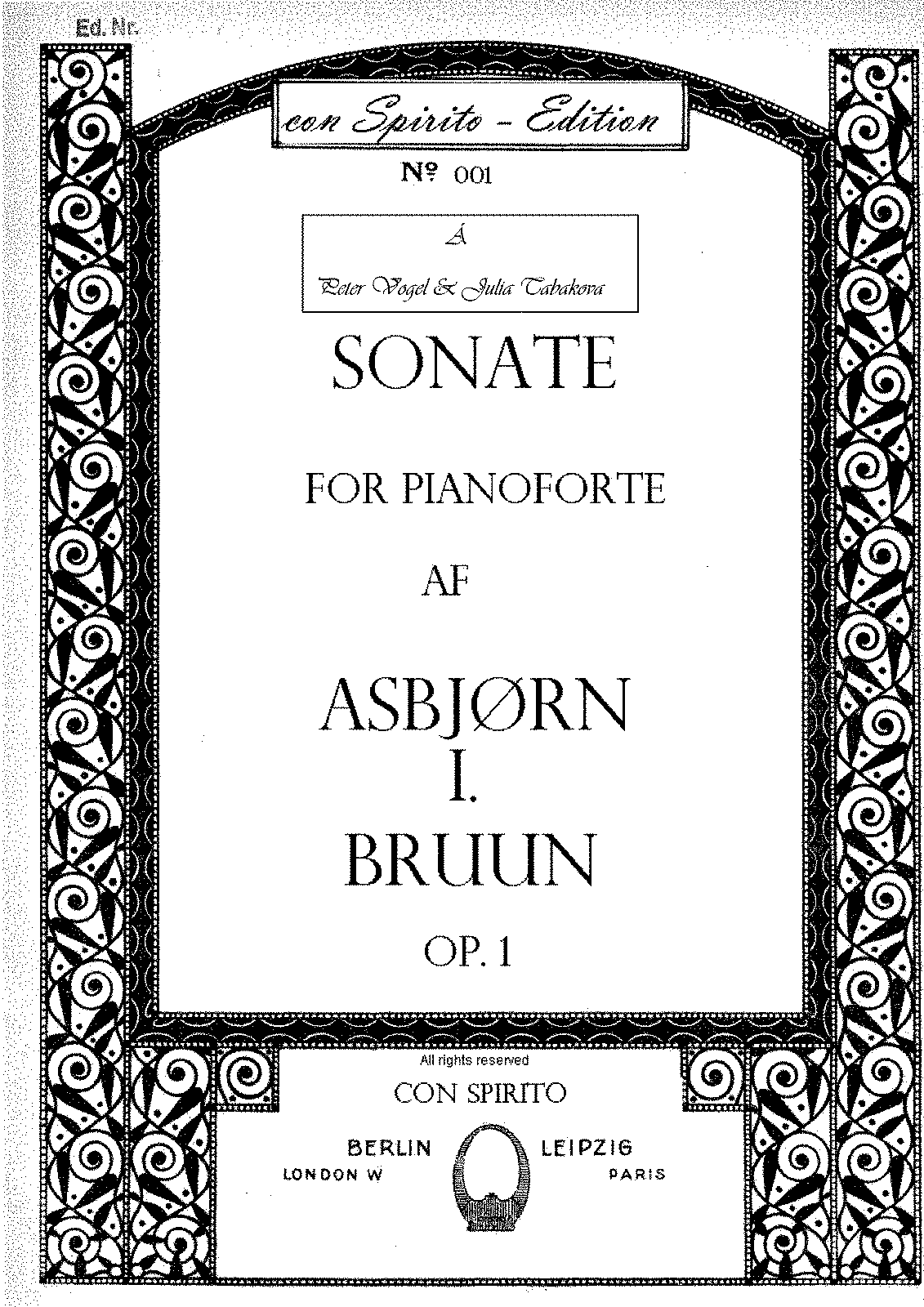

Supplement: S1 Dataset — We obtained the music score images from publicly available music databases, specifically from the IMSLP (International Music Score Library Project). This platform offers a vast collection of music scores that are in the public domain, as well as some modern works with explicit permission. The music score images can be accessed through the following link: https://imslp.org/ The majority of the music score images are sourced from the public domain, and therefore do not have any copyright restrictions. Specifically, the scores selected in our study belong to works in the public domain on the IMSLP platform, which are not protected by current copyright laws (ZIP) [file pone.0323447.s001.zip › PMLP139459.png]

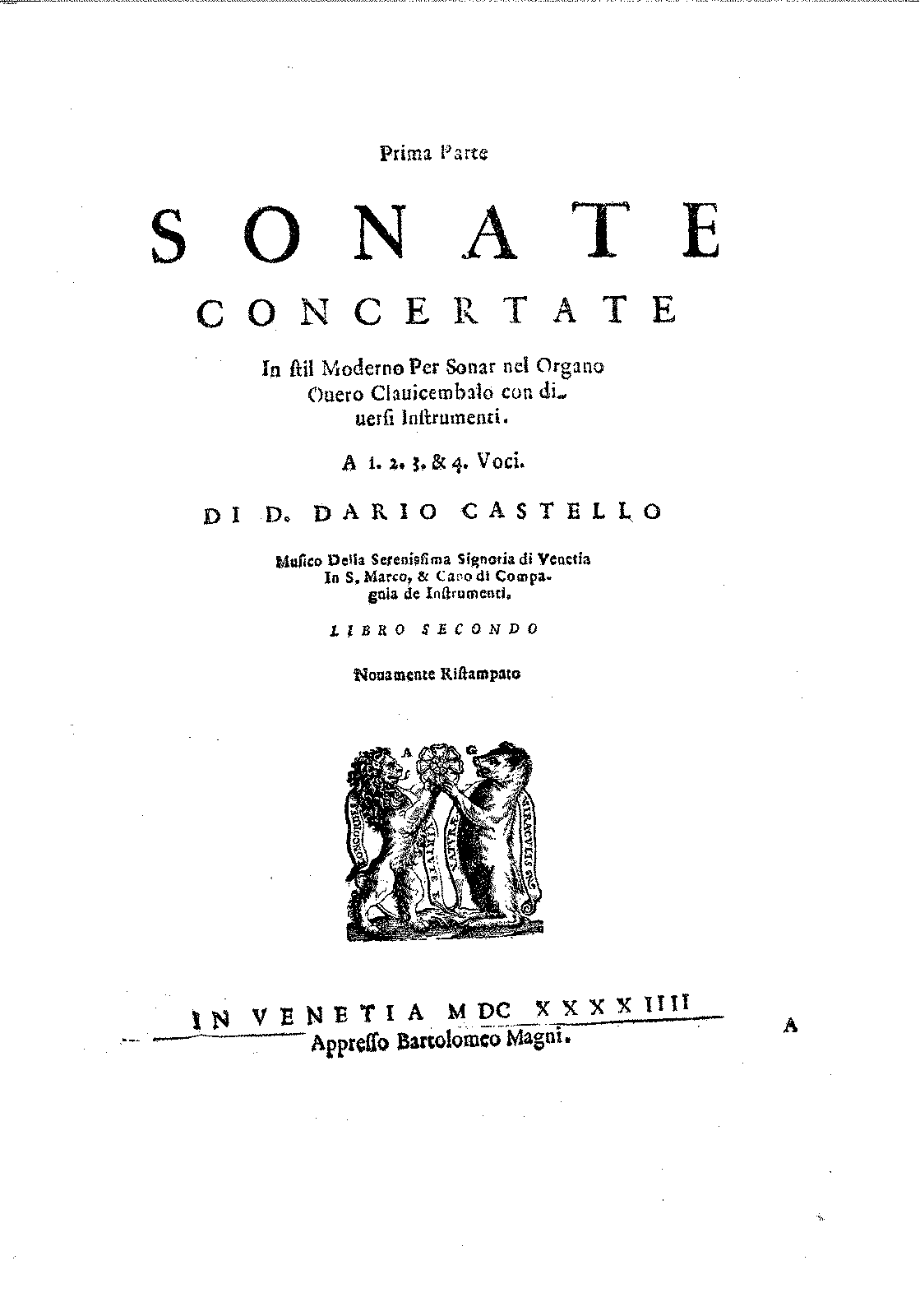

Supplement: S1 Dataset — We obtained the music score images from publicly available music databases, specifically from the IMSLP (International Music Score Library Project). This platform offers a vast collection of music scores that are in the public domain, as well as some modern works with explicit permission. The music score images can be accessed through the following link: https://imslp.org/ The majority of the music score images are sourced from the public domain, and therefore do not have any copyright restrictions. Specifically, the scores selected in our study belong to works in the public domain on the IMSLP platform, which are not protected by current copyright laws (ZIP) [file pone.0323447.s001.zip › PMLP187052.png]

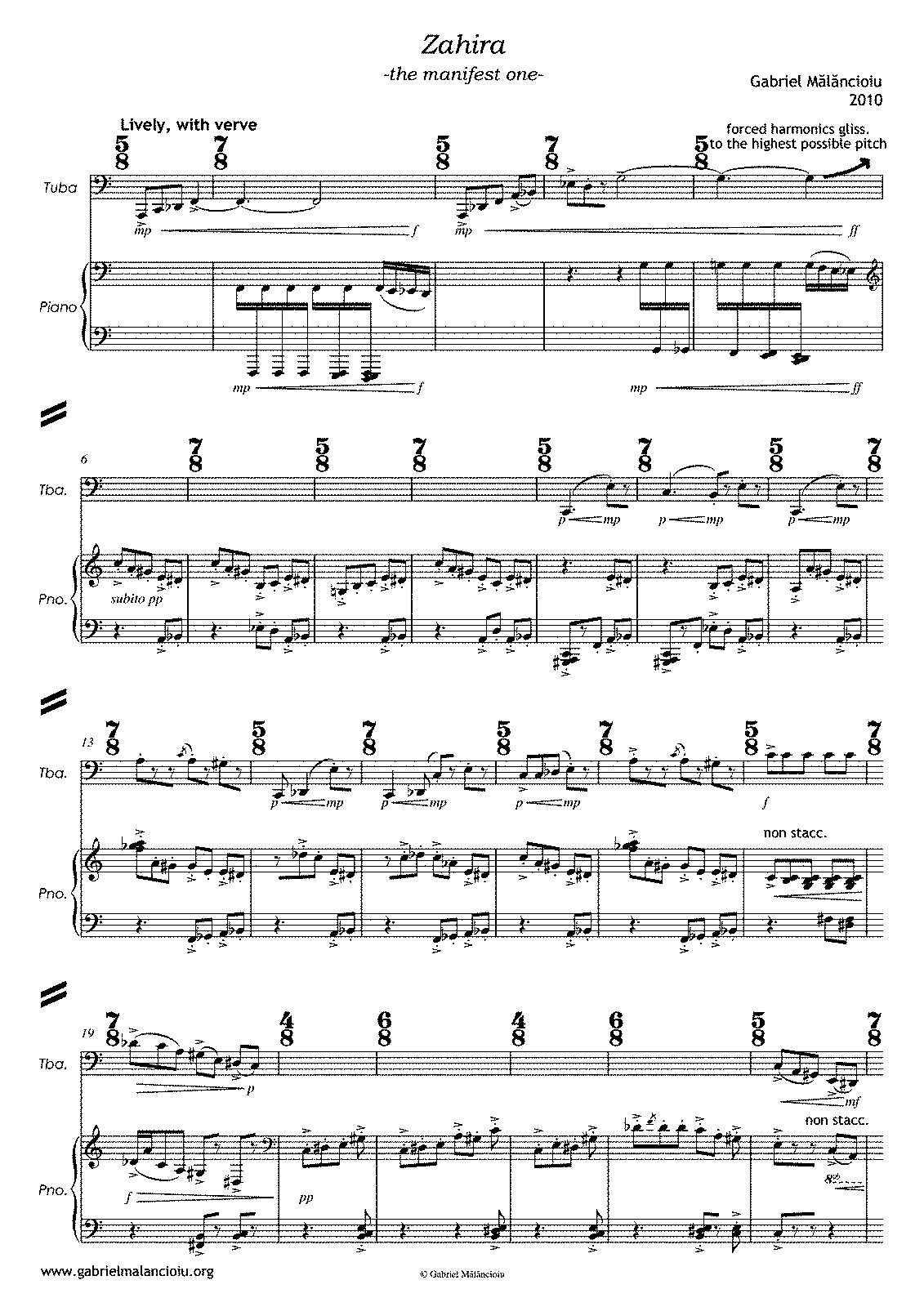

Supplement: S1 Dataset — We obtained the music score images from publicly available music databases, specifically from the IMSLP (International Music Score Library Project). This platform offers a vast collection of music scores that are in the public domain, as well as some modern works with explicit permission. The music score images can be accessed through the following link: https://imslp.org/ The majority of the music score images are sourced from the public domain, and therefore do not have any copyright restrictions. Specifically, the scores selected in our study belong to works in the public domain on the IMSLP platform, which are not protected by current copyright laws (ZIP) [file pone.0323447.s001.zip › PMLP190897.png]

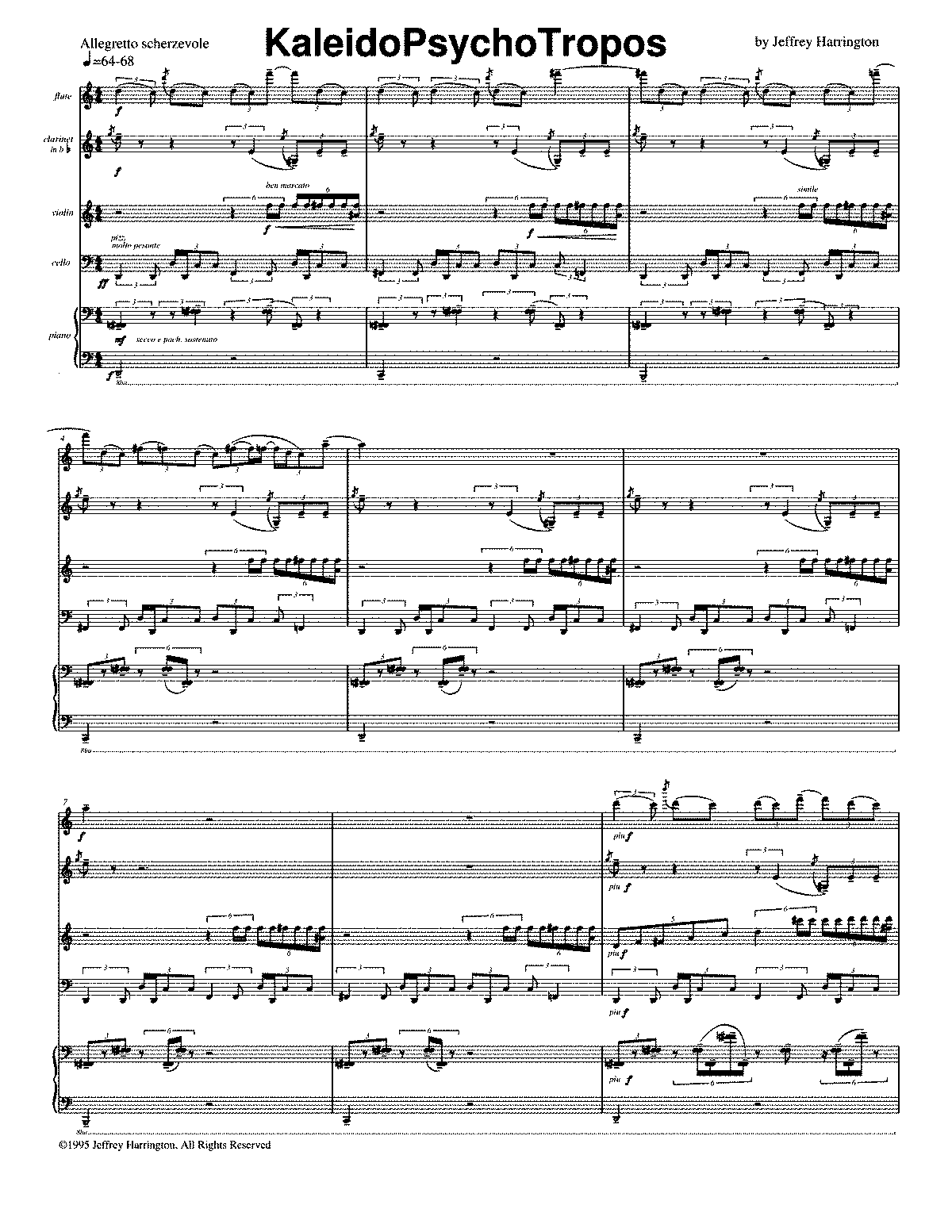

Supplement: S1 Dataset — We obtained the music score images from publicly available music databases, specifically from the IMSLP (International Music Score Library Project). This platform offers a vast collection of music scores that are in the public domain, as well as some modern works with explicit permission. The music score images can be accessed through the following link: https://imslp.org/ The majority of the music score images are sourced from the public domain, and therefore do not have any copyright restrictions. Specifically, the scores selected in our study belong to works in the public domain on the IMSLP platform, which are not protected by current copyright laws (ZIP) [file pone.0323447.s001.zip › PMLP194351.png]

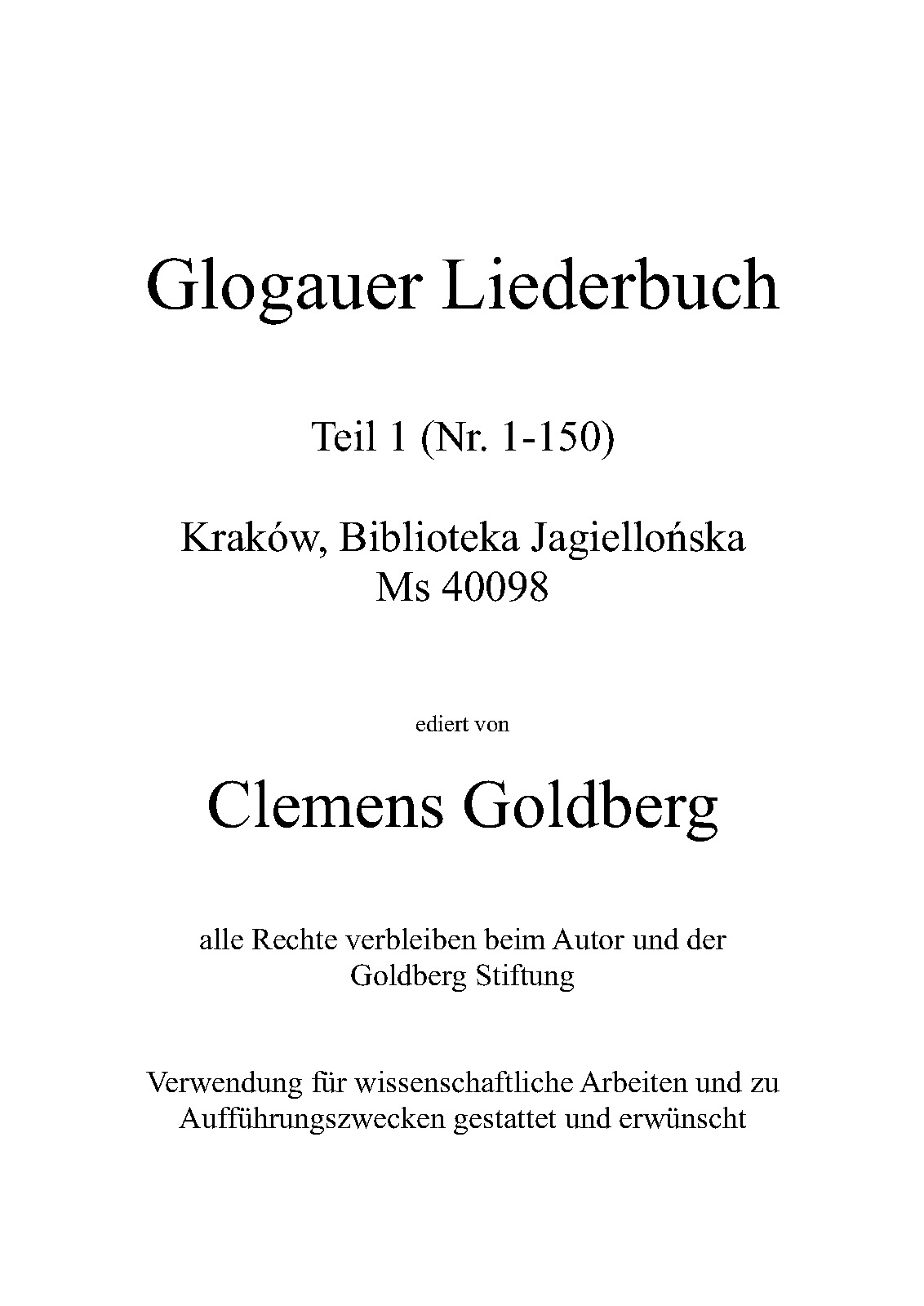

Supplement: S1 Dataset — We obtained the music score images from publicly available music databases, specifically from the IMSLP (International Music Score Library Project). This platform offers a vast collection of music scores that are in the public domain, as well as some modern works with explicit permission. The music score images can be accessed through the following link: https://imslp.org/ The majority of the music score images are sourced from the public domain, and therefore do not have any copyright restrictions. Specifically, the scores selected in our study belong to works in the public domain on the IMSLP platform, which are not protected by current copyright laws (ZIP) [file pone.0323447.s001.zip › PMLP208295.png]

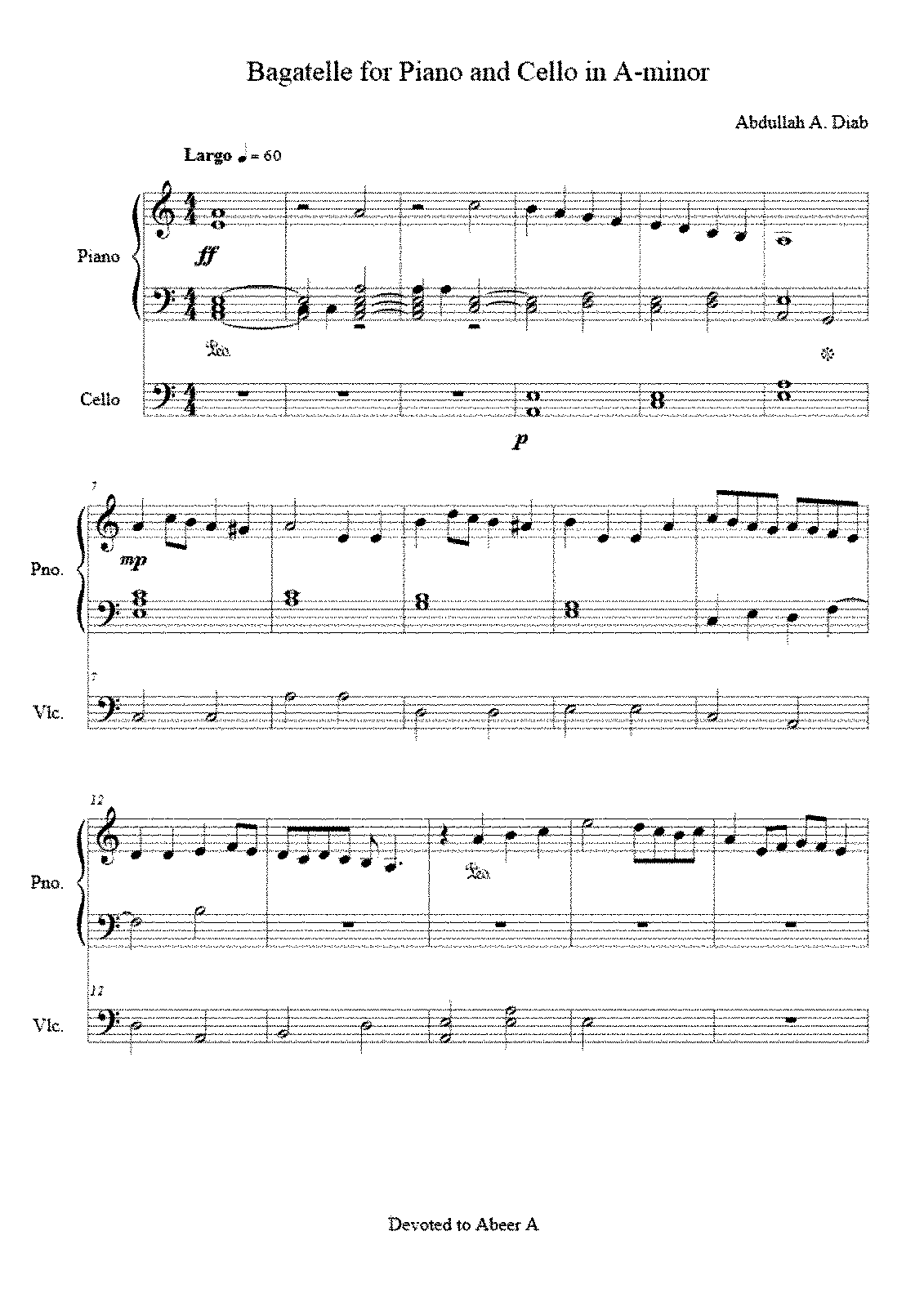

Supplement: S1 Dataset — We obtained the music score images from publicly available music databases, specifically from the IMSLP (International Music Score Library Project). This platform offers a vast collection of music scores that are in the public domain, as well as some modern works with explicit permission. The music score images can be accessed through the following link: https://imslp.org/ The majority of the music score images are sourced from the public domain, and therefore do not have any copyright restrictions. Specifically, the scores selected in our study belong to works in the public domain on the IMSLP platform, which are not protected by current copyright laws (ZIP) [file pone.0323447.s001.zip › PMLP272437.png]

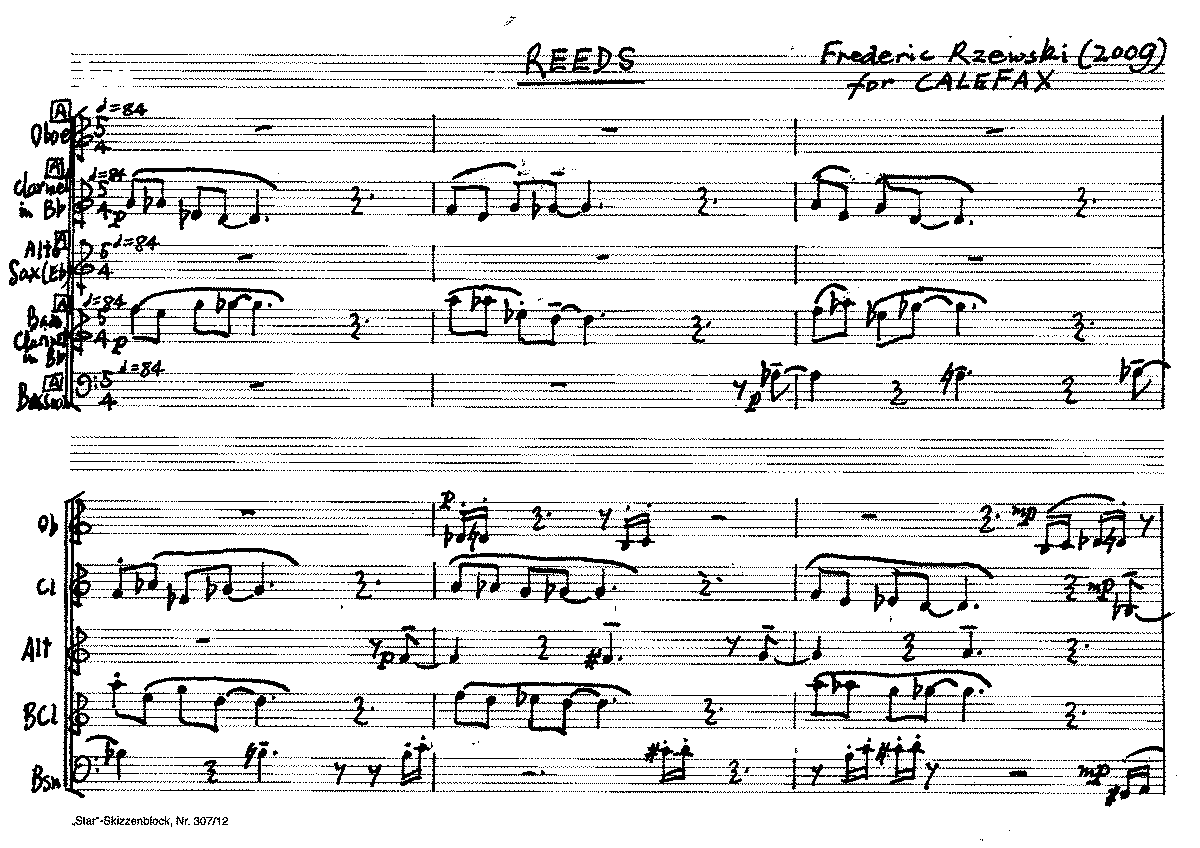

Supplement: S1 Dataset — We obtained the music score images from publicly available music databases, specifically from the IMSLP (International Music Score Library Project). This platform offers a vast collection of music scores that are in the public domain, as well as some modern works with explicit permission. The music score images can be accessed through the following link: https://imslp.org/ The majority of the music score images are sourced from the public domain, and therefore do not have any copyright restrictions. Specifically, the scores selected in our study belong to works in the public domain on the IMSLP platform, which are not protected by current copyright laws (ZIP) [file pone.0323447.s001.zip › PMLP282541.png]

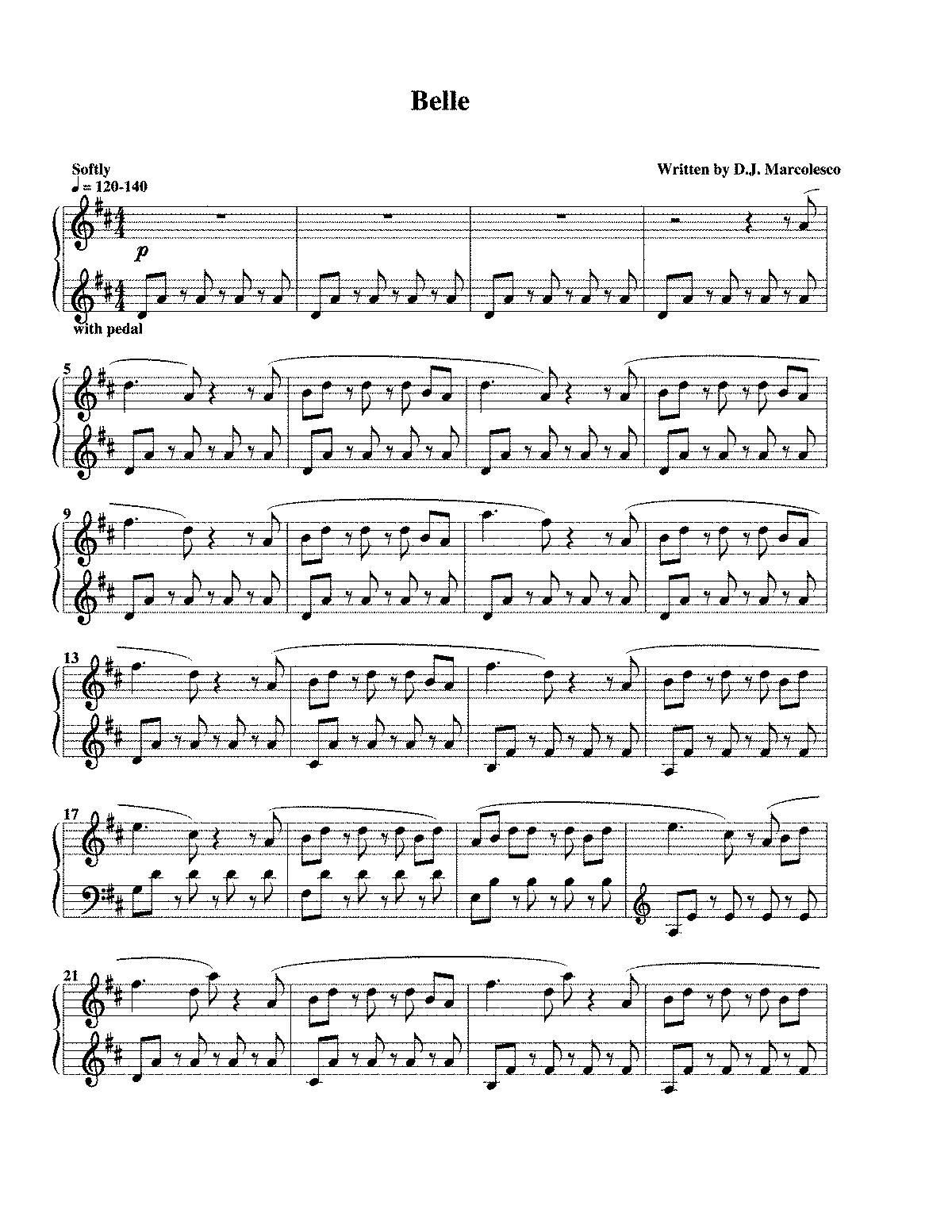

Supplement: S1 Dataset — We obtained the music score images from publicly available music databases, specifically from the IMSLP (International Music Score Library Project). This platform offers a vast collection of music scores that are in the public domain, as well as some modern works with explicit permission. The music score images can be accessed through the following link: https://imslp.org/ The majority of the music score images are sourced from the public domain, and therefore do not have any copyright restrictions. Specifically, the scores selected in our study belong to works in the public domain on the IMSLP platform, which are not protected by current copyright laws (ZIP) [file pone.0323447.s001.zip › PMLP287269.png]

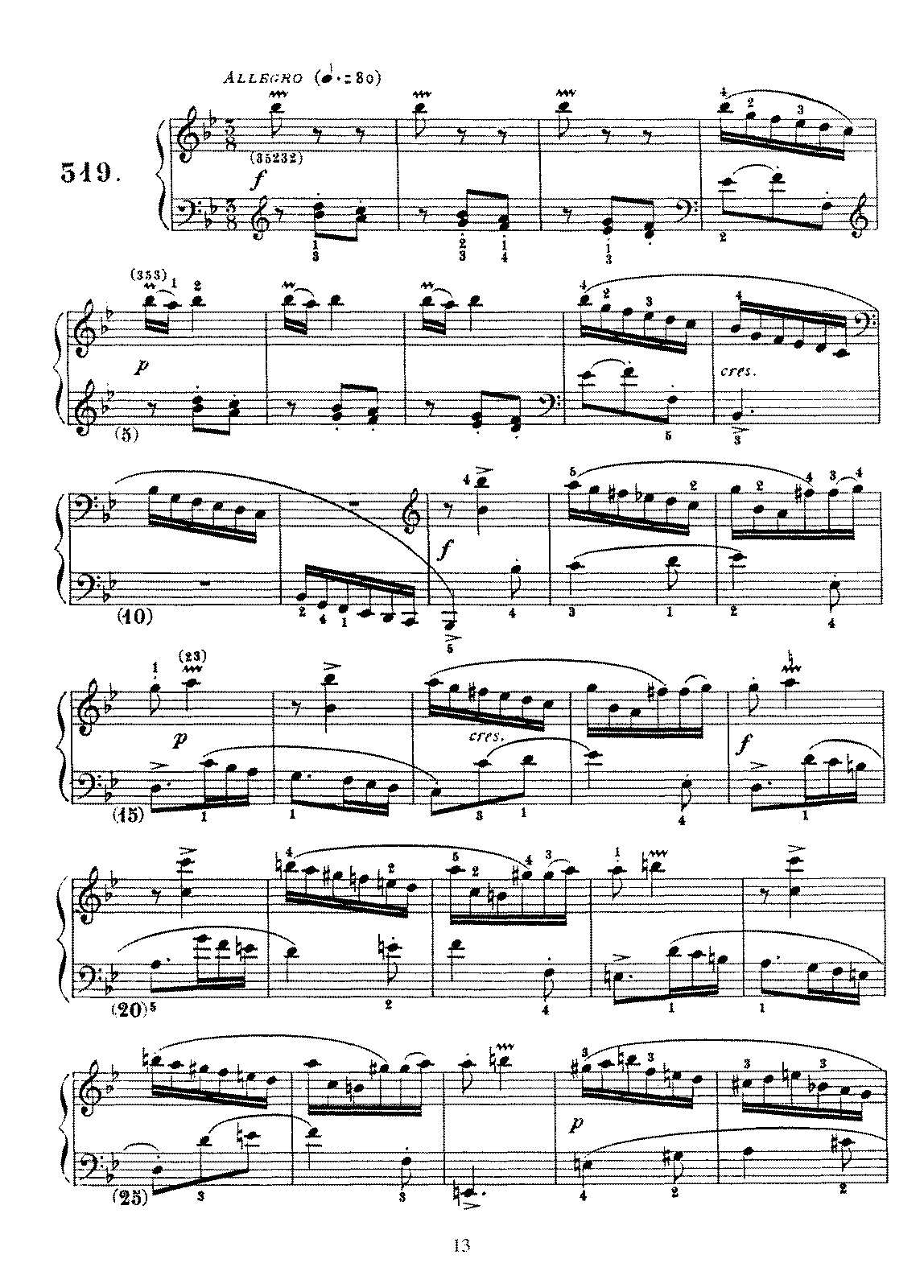

Supplement: S1 Dataset — We obtained the music score images from publicly available music databases, specifically from the IMSLP (International Music Score Library Project). This platform offers a vast collection of music scores that are in the public domain, as well as some modern works with explicit permission. The music score images can be accessed through the following link: https://imslp.org/ The majority of the music score images are sourced from the public domain, and therefore do not have any copyright restrictions. Specifically, the scores selected in our study belong to works in the public domain on the IMSLP platform, which are not protected by current copyright laws (ZIP) [file pone.0323447.s001.zip › PMLP297356.png]

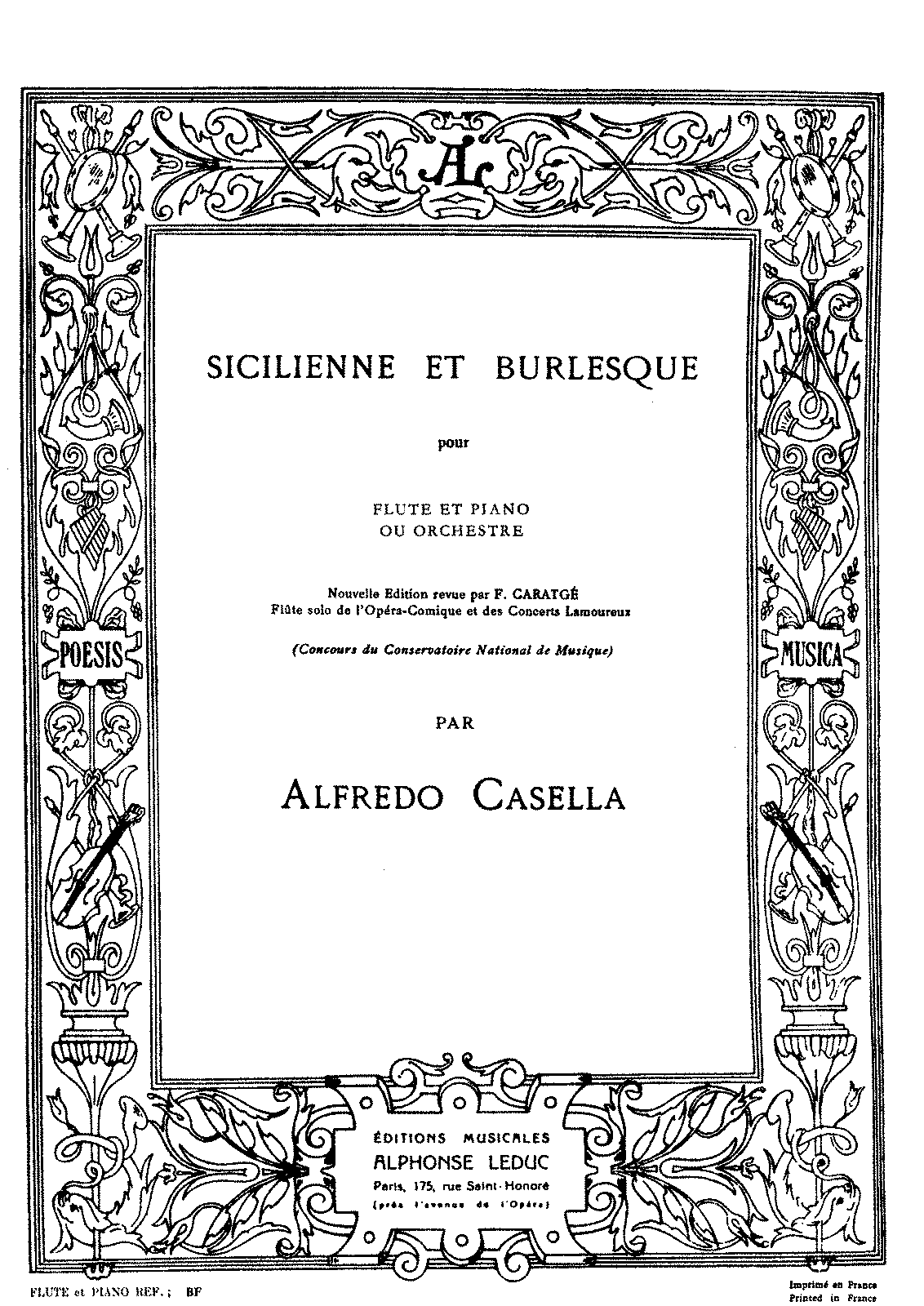

Supplement: S1 Dataset — We obtained the music score images from publicly available music databases, specifically from the IMSLP (International Music Score Library Project). This platform offers a vast collection of music scores that are in the public domain, as well as some modern works with explicit permission. The music score images can be accessed through the following link: https://imslp.org/ The majority of the music score images are sourced from the public domain, and therefore do not have any copyright restrictions. Specifically, the scores selected in our study belong to works in the public domain on the IMSLP platform, which are not protected by current copyright laws (ZIP) [file pone.0323447.s001.zip › PMLP299166.png]

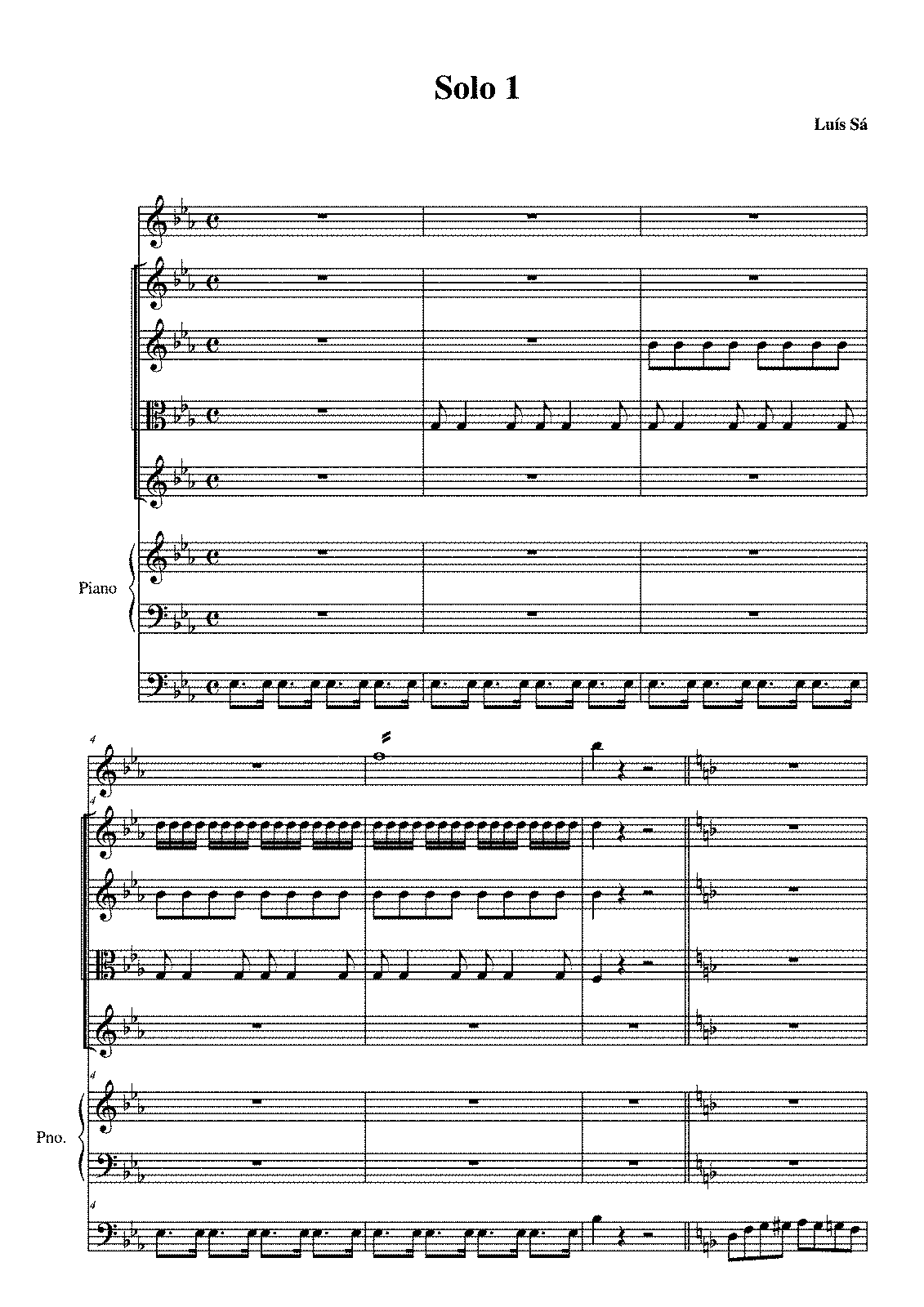

Supplement: S1 Dataset — We obtained the music score images from publicly available music databases, specifically from the IMSLP (International Music Score Library Project). This platform offers a vast collection of music scores that are in the public domain, as well as some modern works with explicit permission. The music score images can be accessed through the following link: https://imslp.org/ The majority of the music score images are sourced from the public domain, and therefore do not have any copyright restrictions. Specifically, the scores selected in our study belong to works in the public domain on the IMSLP platform, which are not protected by current copyright laws (ZIP) [file pone.0323447.s001.zip › PMLP301739.png]

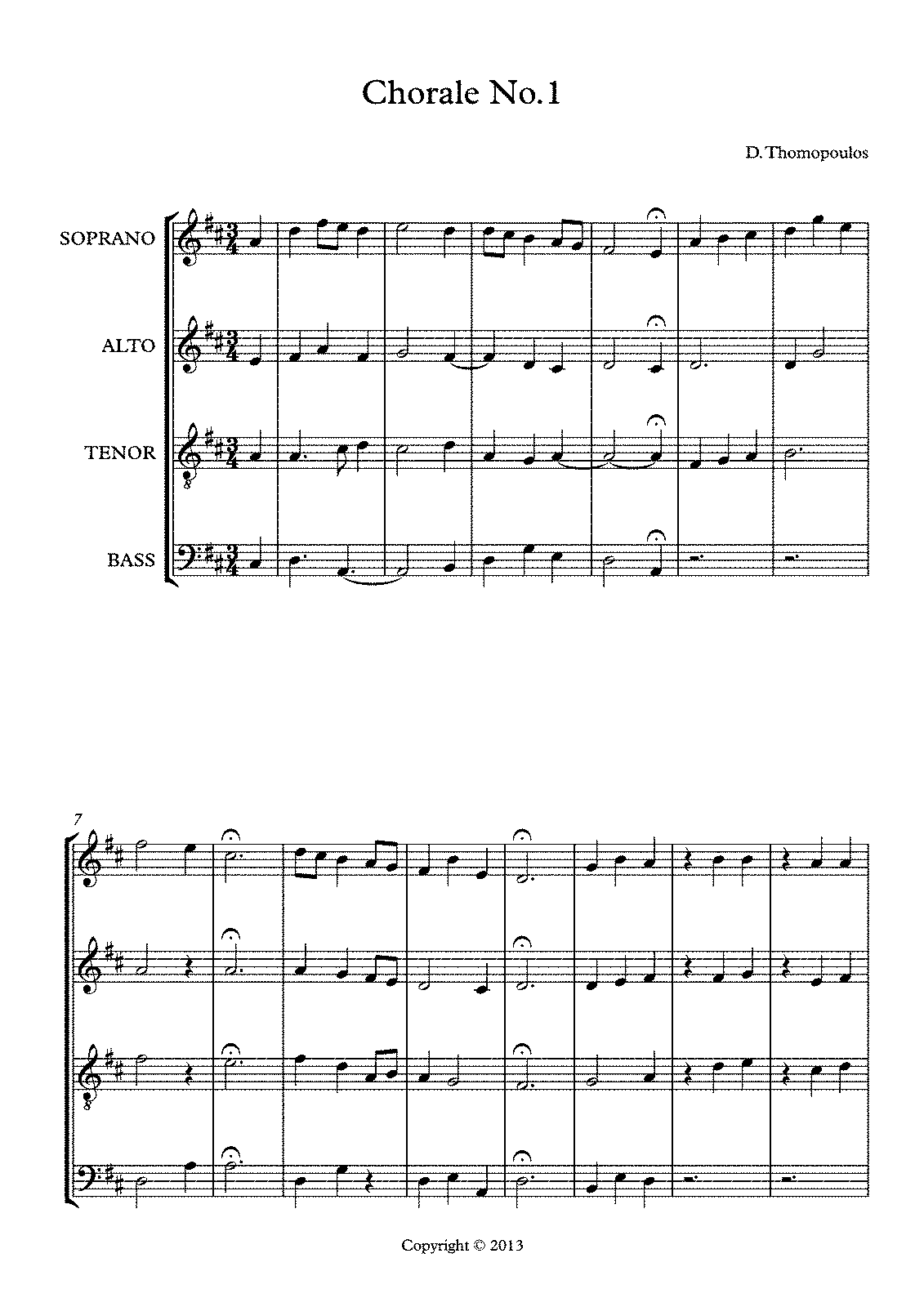

Supplement: S1 Dataset — We obtained the music score images from publicly available music databases, specifically from the IMSLP (International Music Score Library Project). This platform offers a vast collection of music scores that are in the public domain, as well as some modern works with explicit permission. The music score images can be accessed through the following link: https://imslp.org/ The majority of the music score images are sourced from the public domain, and therefore do not have any copyright restrictions. Specifically, the scores selected in our study belong to works in the public domain on the IMSLP platform, which are not protected by current copyright laws (ZIP) [file pone.0323447.s001.zip › PMLP430330.png]

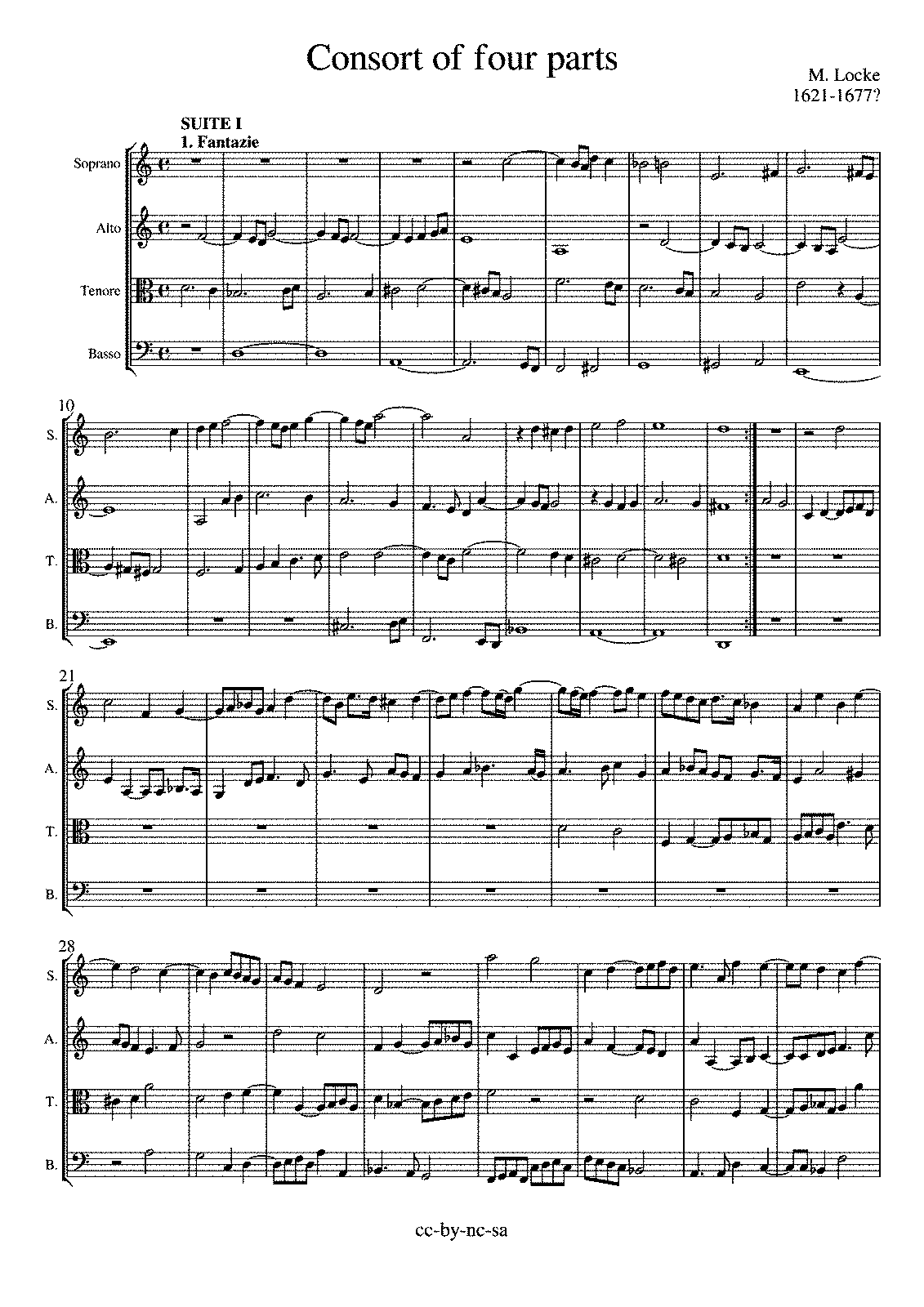

Supplement: S1 Dataset — We obtained the music score images from publicly available music databases, specifically from the IMSLP (International Music Score Library Project). This platform offers a vast collection of music scores that are in the public domain, as well as some modern works with explicit permission. The music score images can be accessed through the following link: https://imslp.org/ The majority of the music score images are sourced from the public domain, and therefore do not have any copyright restrictions. Specifically, the scores selected in our study belong to works in the public domain on the IMSLP platform, which are not protected by current copyright laws (ZIP) [file pone.0323447.s001.zip › PMLP432014.png]

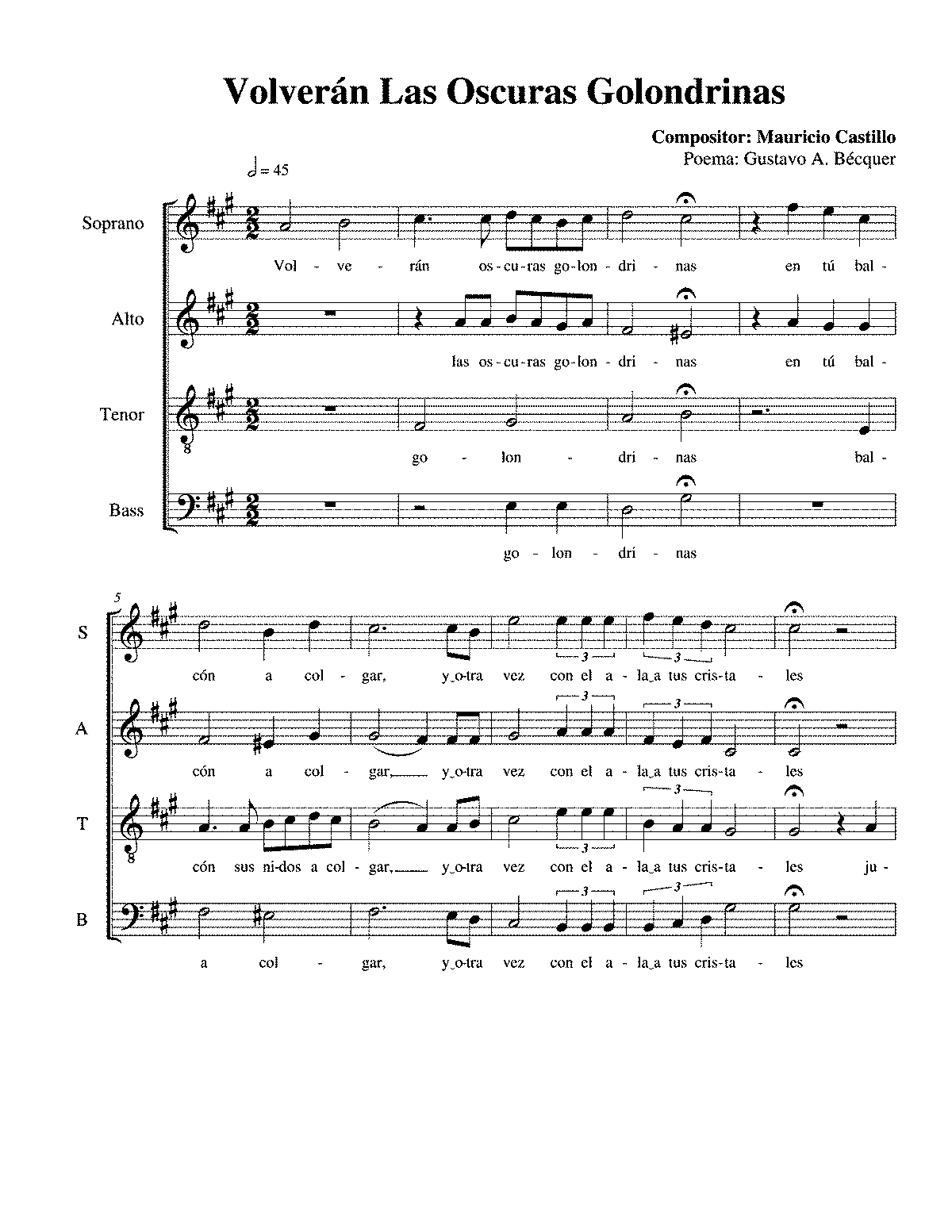

Supplement: S1 Dataset — We obtained the music score images from publicly available music databases, specifically from the IMSLP (International Music Score Library Project). This platform offers a vast collection of music scores that are in the public domain, as well as some modern works with explicit permission. The music score images can be accessed through the following link: https://imslp.org/ The majority of the music score images are sourced from the public domain, and therefore do not have any copyright restrictions. Specifically, the scores selected in our study belong to works in the public domain on the IMSLP platform, which are not protected by current copyright laws (ZIP) [file pone.0323447.s001.zip › PMLP432597.png]

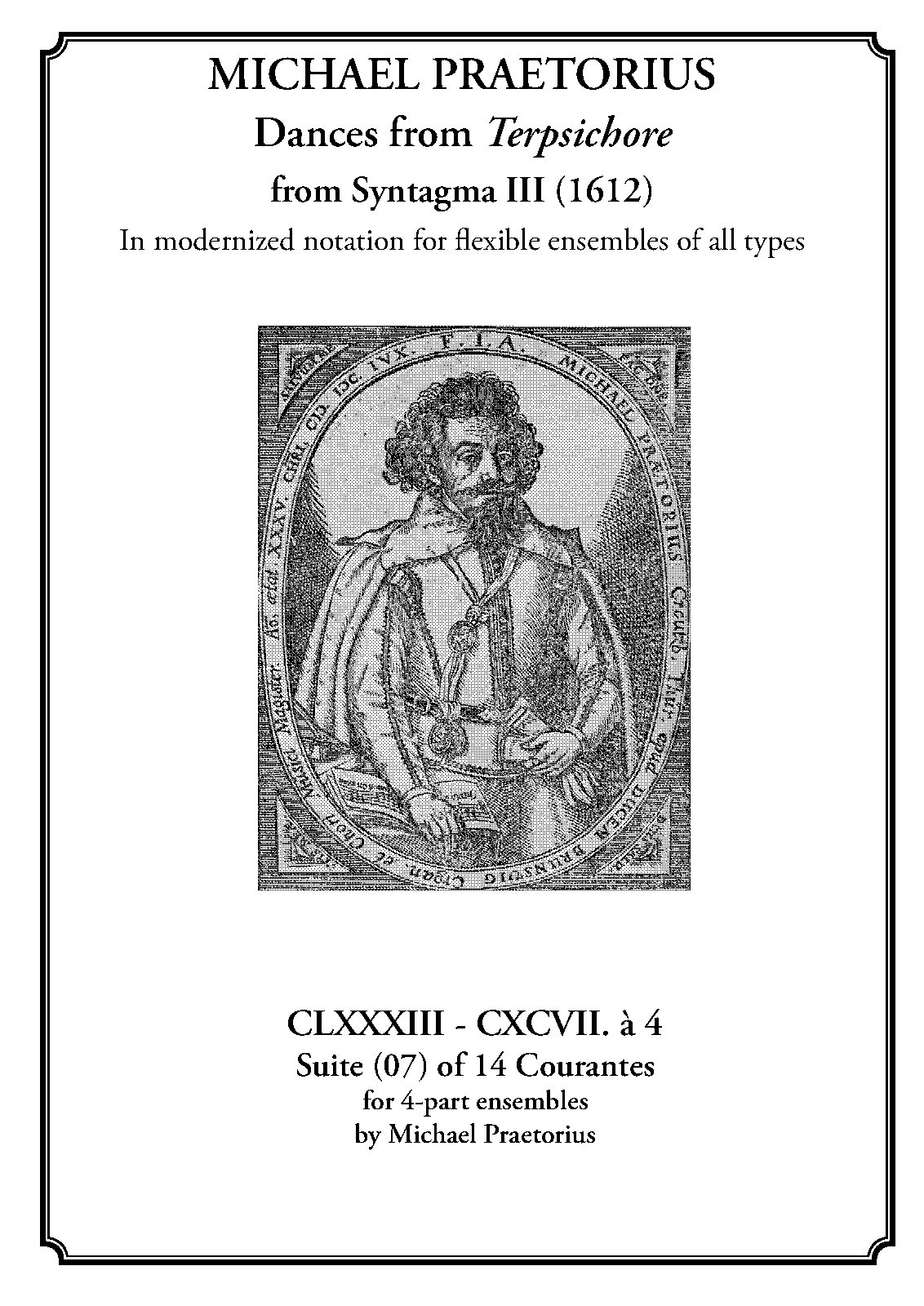

Supplement: S1 Dataset — We obtained the music score images from publicly available music databases, specifically from the IMSLP (International Music Score Library Project). This platform offers a vast collection of music scores that are in the public domain, as well as some modern works with explicit permission. The music score images can be accessed through the following link: https://imslp.org/ The majority of the music score images are sourced from the public domain, and therefore do not have any copyright restrictions. Specifically, the scores selected in our study belong to works in the public domain on the IMSLP platform, which are not protected by current copyright laws (ZIP) [file pone.0323447.s001.zip › PMLP598949.png]

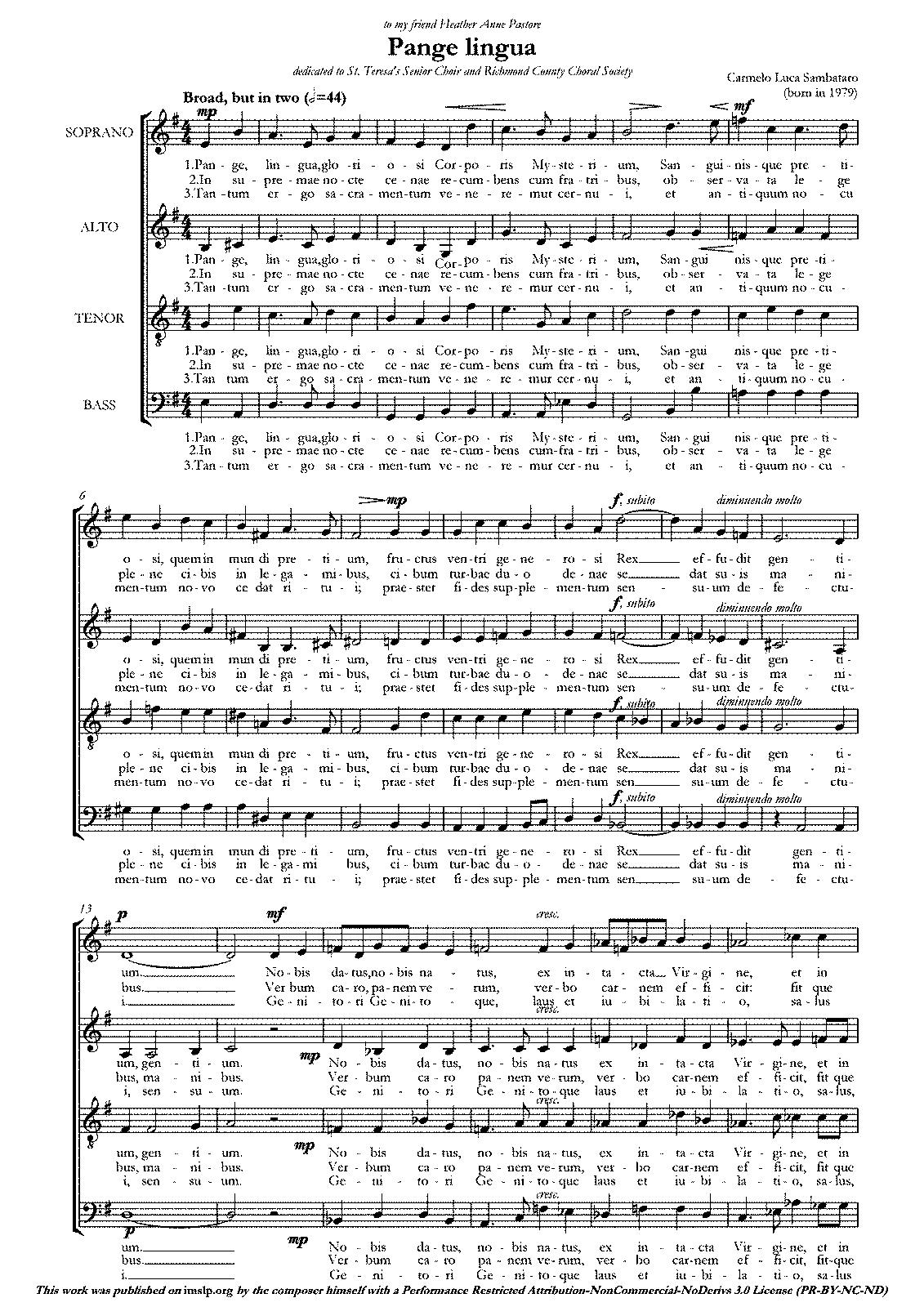

Supplement: S1 Dataset — We obtained the music score images from publicly available music databases, specifically from the IMSLP (International Music Score Library Project). This platform offers a vast collection of music scores that are in the public domain, as well as some modern works with explicit permission. The music score images can be accessed through the following link: https://imslp.org/ The majority of the music score images are sourced from the public domain, and therefore do not have any copyright restrictions. Specifically, the scores selected in our study belong to works in the public domain on the IMSLP platform, which are not protected by current copyright laws (ZIP) [file pone.0323447.s001.zip › PMLP599239.png]

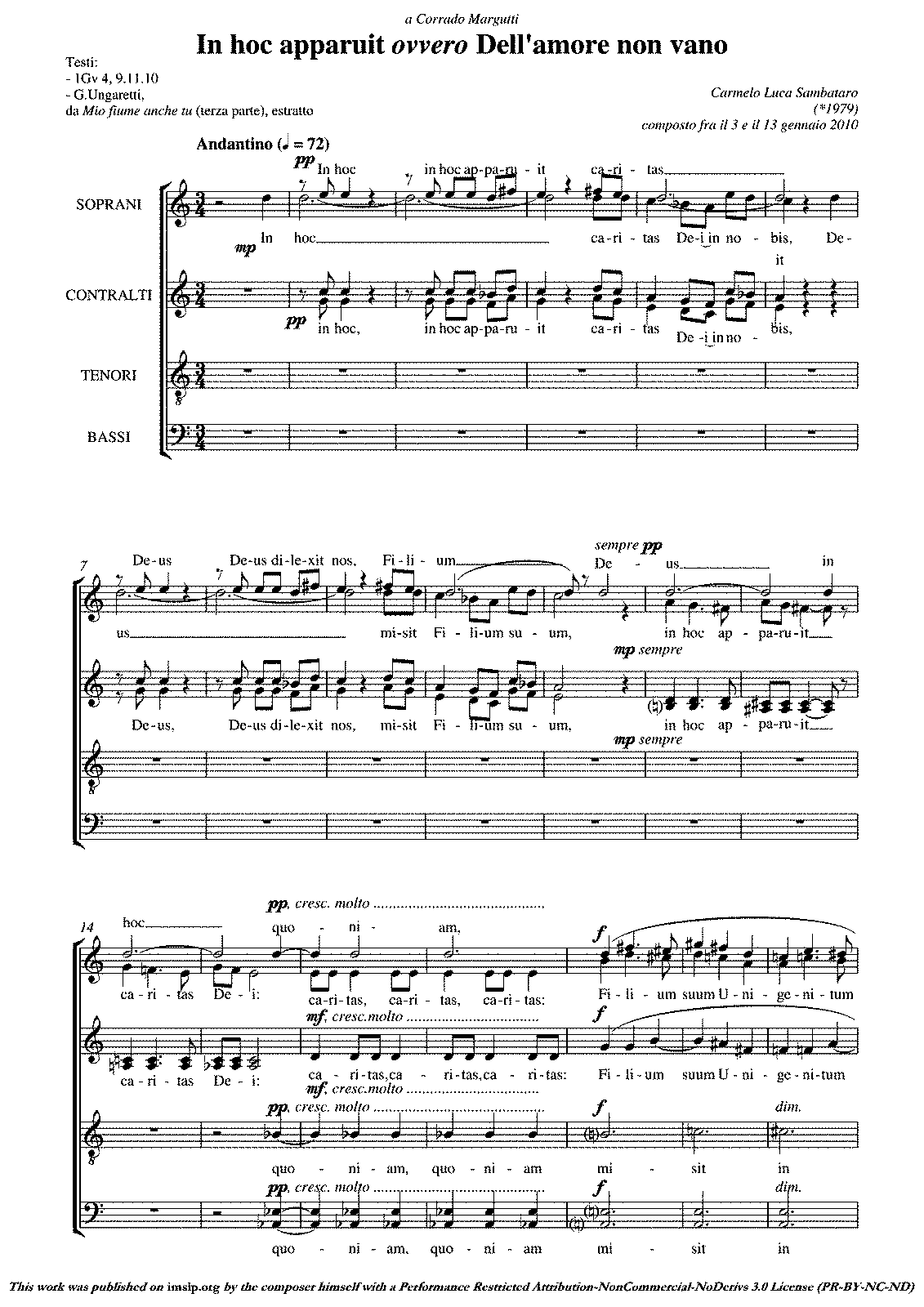

Supplement: S1 Dataset — We obtained the music score images from publicly available music databases, specifically from the IMSLP (International Music Score Library Project). This platform offers a vast collection of music scores that are in the public domain, as well as some modern works with explicit permission. The music score images can be accessed through the following link: https://imslp.org/ The majority of the music score images are sourced from the public domain, and therefore do not have any copyright restrictions. Specifically, the scores selected in our study belong to works in the public domain on the IMSLP platform, which are not protected by current copyright laws (ZIP) [file pone.0323447.s001.zip › PMLP599246.png]

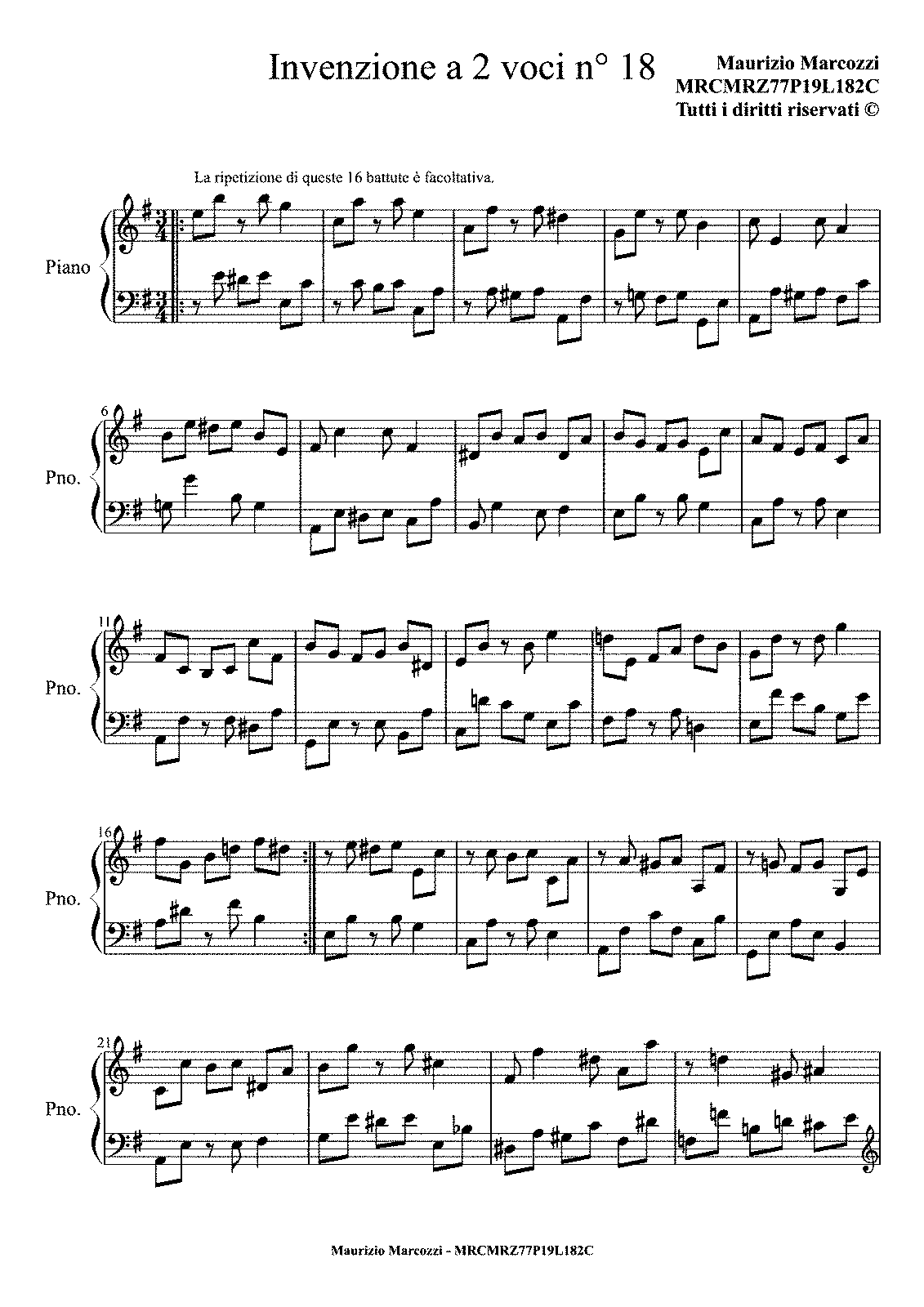

Supplement: S1 Dataset — We obtained the music score images from publicly available music databases, specifically from the IMSLP (International Music Score Library Project). This platform offers a vast collection of music scores that are in the public domain, as well as some modern works with explicit permission. The music score images can be accessed through the following link: https://imslp.org/ The majority of the music score images are sourced from the public domain, and therefore do not have any copyright restrictions. Specifically, the scores selected in our study belong to works in the public domain on the IMSLP platform, which are not protected by current copyright laws (ZIP) [file pone.0323447.s001.zip › PMLP617350.png]

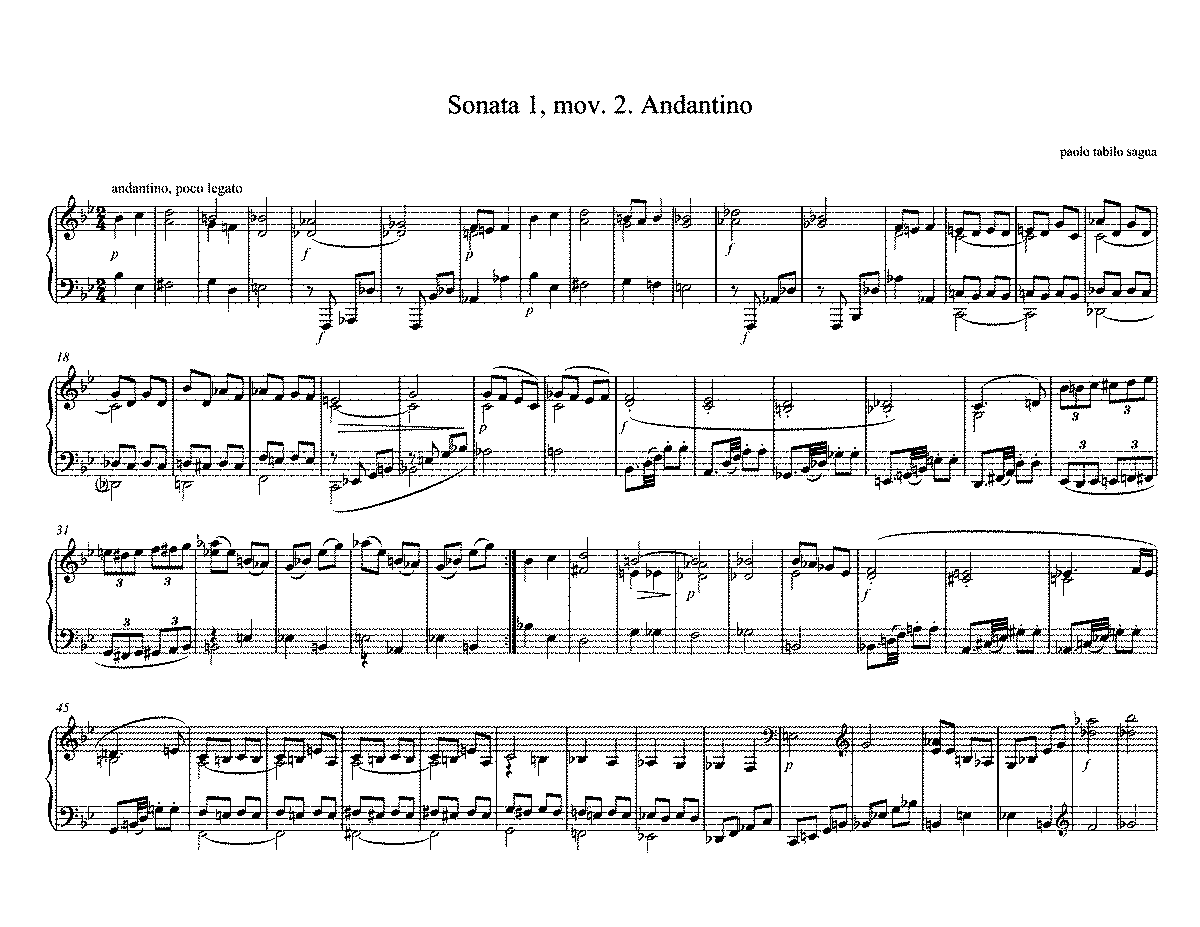

Supplement: S1 Dataset — We obtained the music score images from publicly available music databases, specifically from the IMSLP (International Music Score Library Project). This platform offers a vast collection of music scores that are in the public domain, as well as some modern works with explicit permission. The music score images can be accessed through the following link: https://imslp.org/ The majority of the music score images are sourced from the public domain, and therefore do not have any copyright restrictions. Specifically, the scores selected in our study belong to works in the public domain on the IMSLP platform, which are not protected by current copyright laws (ZIP) [file pone.0323447.s001.zip › PMLP618608.png]

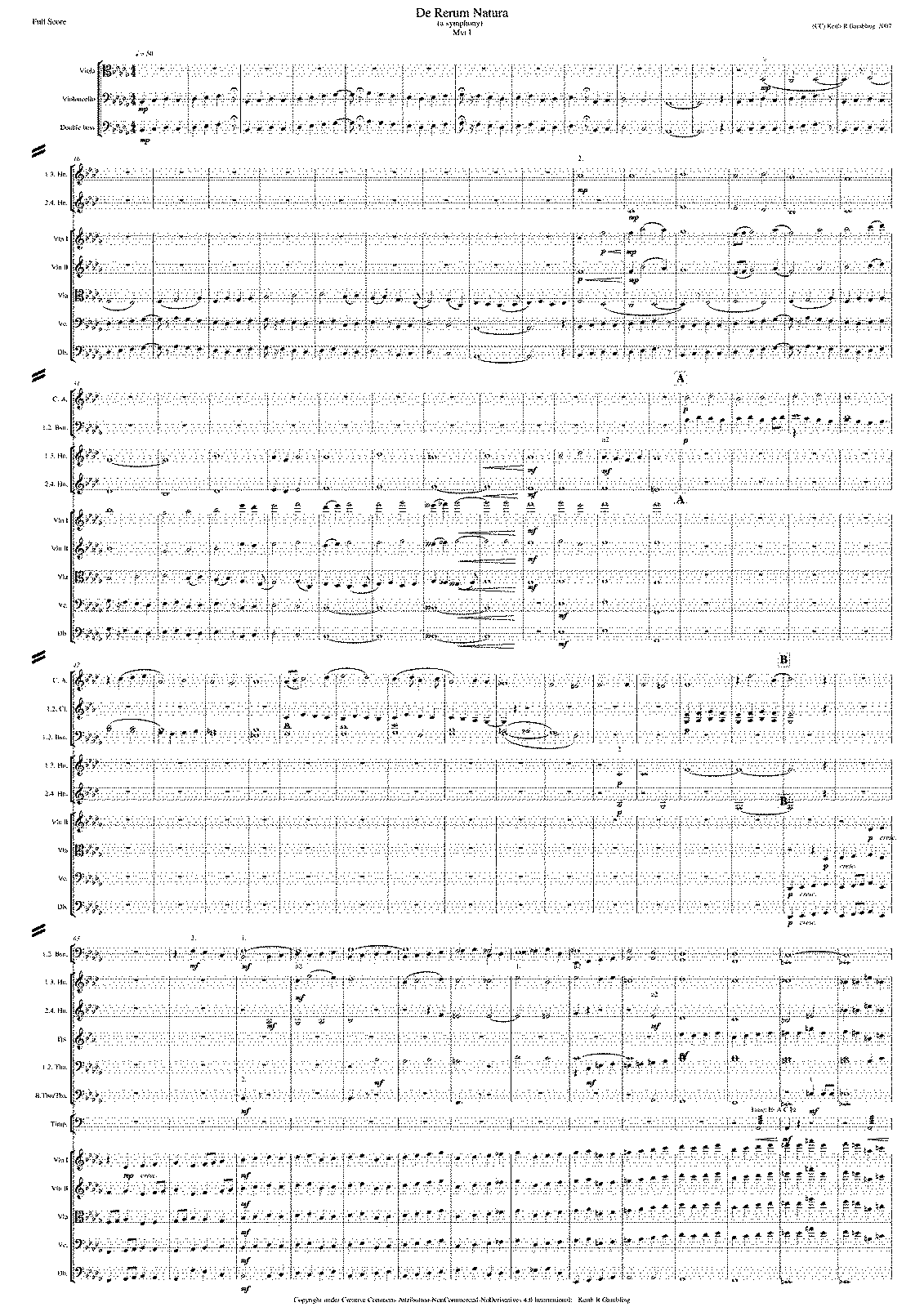

Supplement: S1 Dataset — We obtained the music score images from publicly available music databases, specifically from the IMSLP (International Music Score Library Project). This platform offers a vast collection of music scores that are in the public domain, as well as some modern works with explicit permission. The music score images can be accessed through the following link: https://imslp.org/ The majority of the music score images are sourced from the public domain, and therefore do not have any copyright restrictions. Specifically, the scores selected in our study belong to works in the public domain on the IMSLP platform, which are not protected by current copyright laws (ZIP) [file pone.0323447.s001.zip › PMLP633305.png]

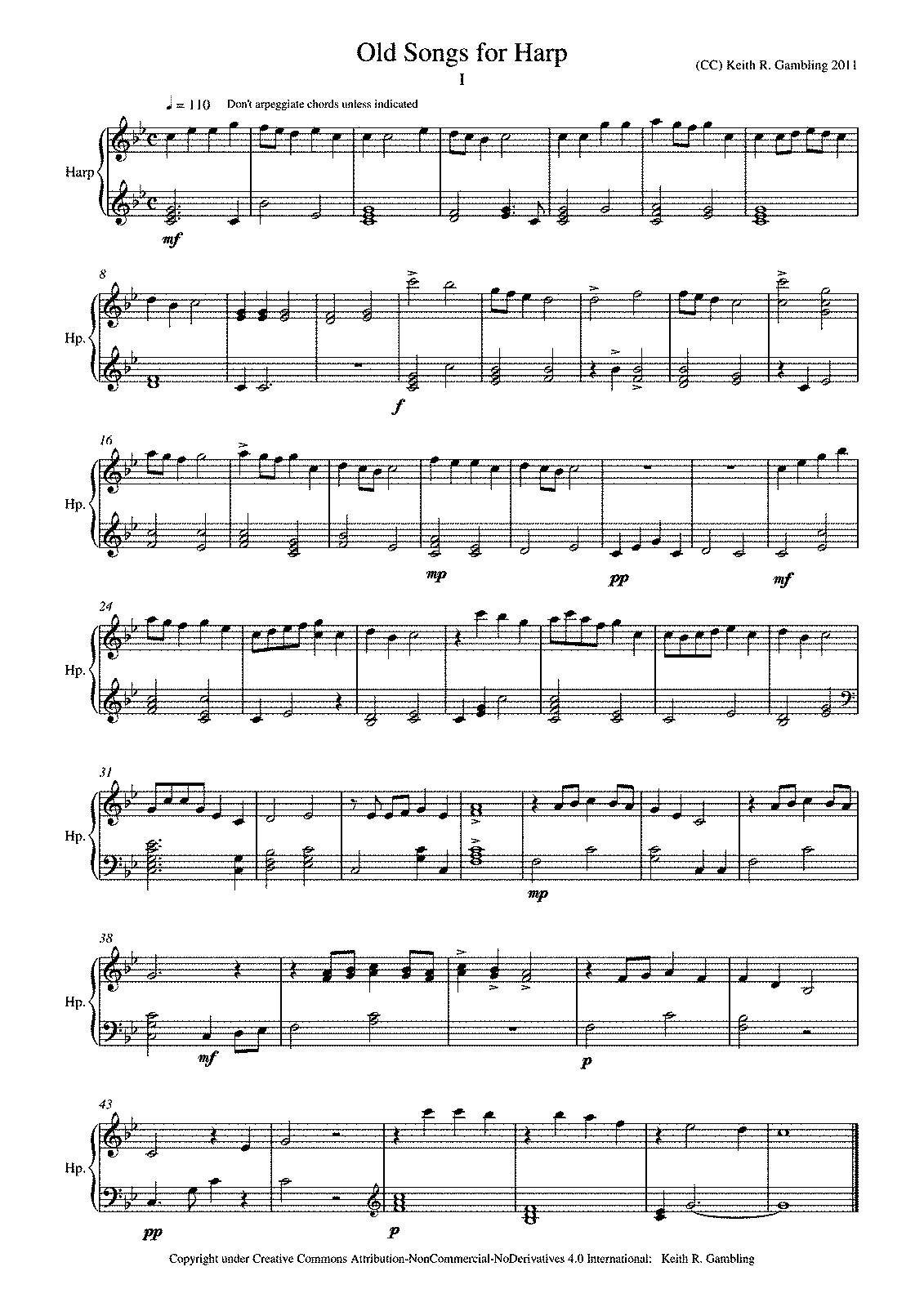

Supplement: S1 Dataset — We obtained the music score images from publicly available music databases, specifically from the IMSLP (International Music Score Library Project). This platform offers a vast collection of music scores that are in the public domain, as well as some modern works with explicit permission. The music score images can be accessed through the following link: https://imslp.org/ The majority of the music score images are sourced from the public domain, and therefore do not have any copyright restrictions. Specifically, the scores selected in our study belong to works in the public domain on the IMSLP platform, which are not protected by current copyright laws (ZIP) [file pone.0323447.s001.zip › PMLP636920.png]

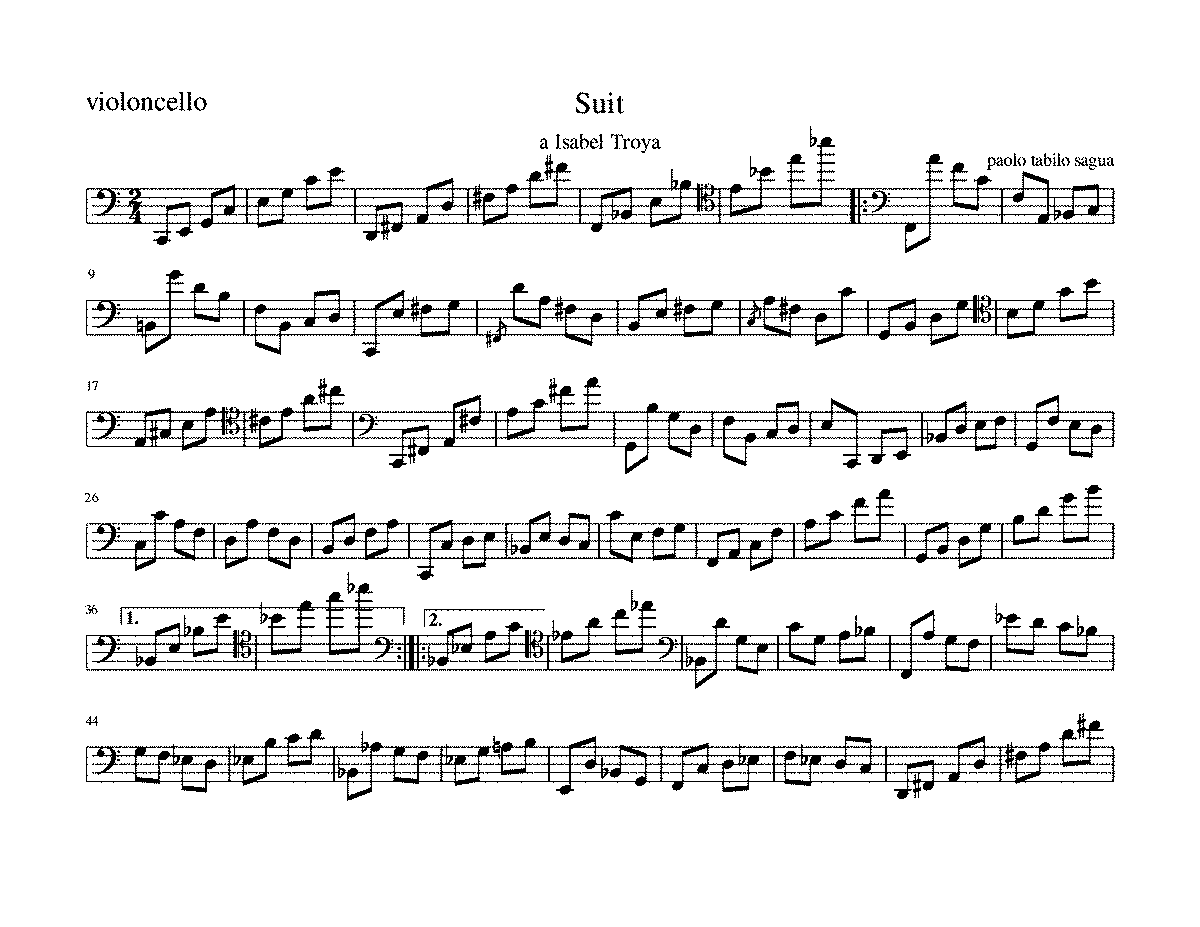

Supplement: S1 Dataset — We obtained the music score images from publicly available music databases, specifically from the IMSLP (International Music Score Library Project). This platform offers a vast collection of music scores that are in the public domain, as well as some modern works with explicit permission. The music score images can be accessed through the following link: https://imslp.org/ The majority of the music score images are sourced from the public domain, and therefore do not have any copyright restrictions. Specifically, the scores selected in our study belong to works in the public domain on the IMSLP platform, which are not protected by current copyright laws (ZIP) [file pone.0323447.s001.zip › PMLP796075.png]

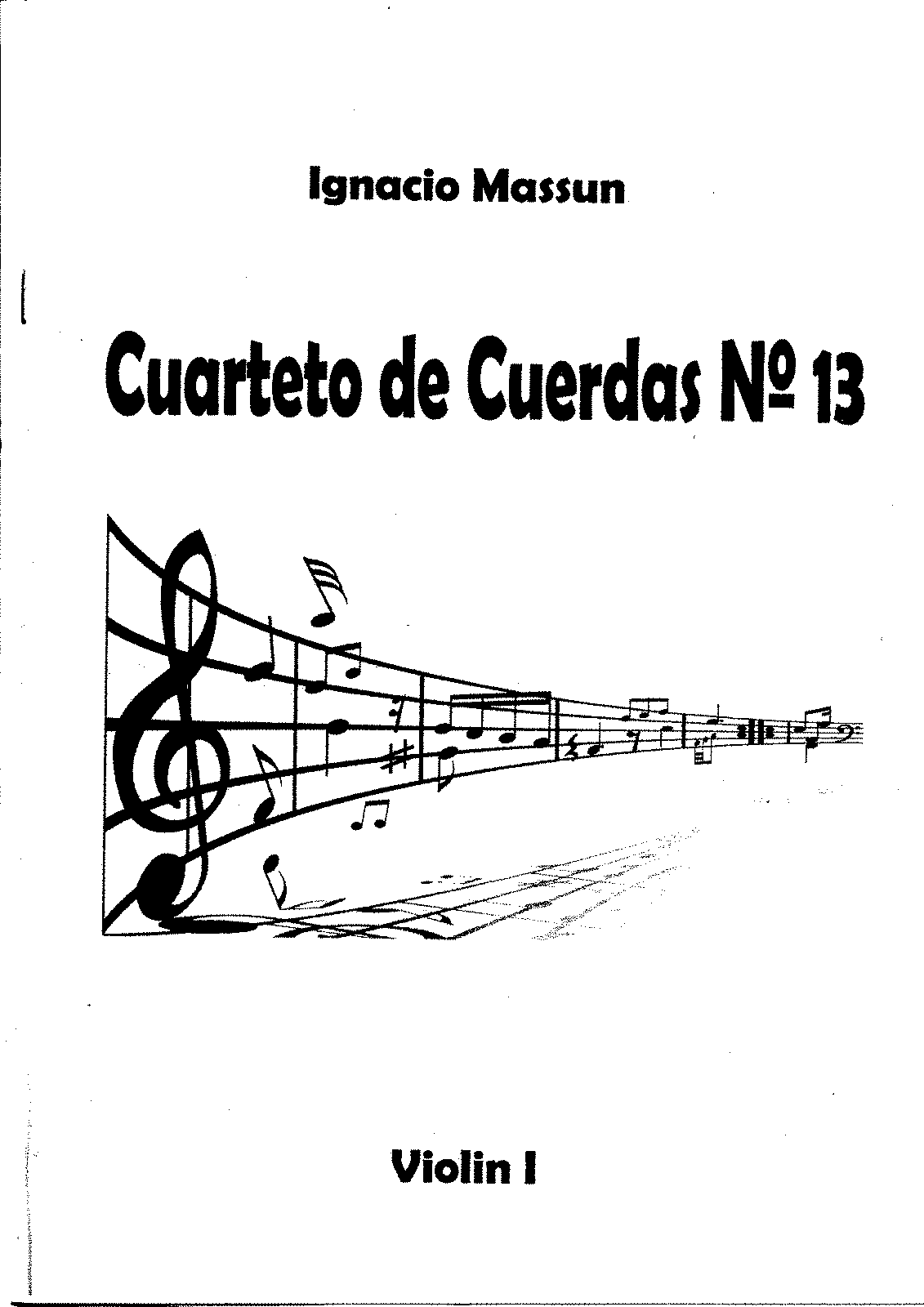

Supplement: S1 Dataset — We obtained the music score images from publicly available music databases, specifically from the IMSLP (International Music Score Library Project). This platform offers a vast collection of music scores that are in the public domain, as well as some modern works with explicit permission. The music score images can be accessed through the following link: https://imslp.org/ The majority of the music score images are sourced from the public domain, and therefore do not have any copyright restrictions. Specifically, the scores selected in our study belong to works in the public domain on the IMSLP platform, which are not protected by current copyright laws (ZIP) [file pone.0323447.s001.zip › PMLP924484.png]

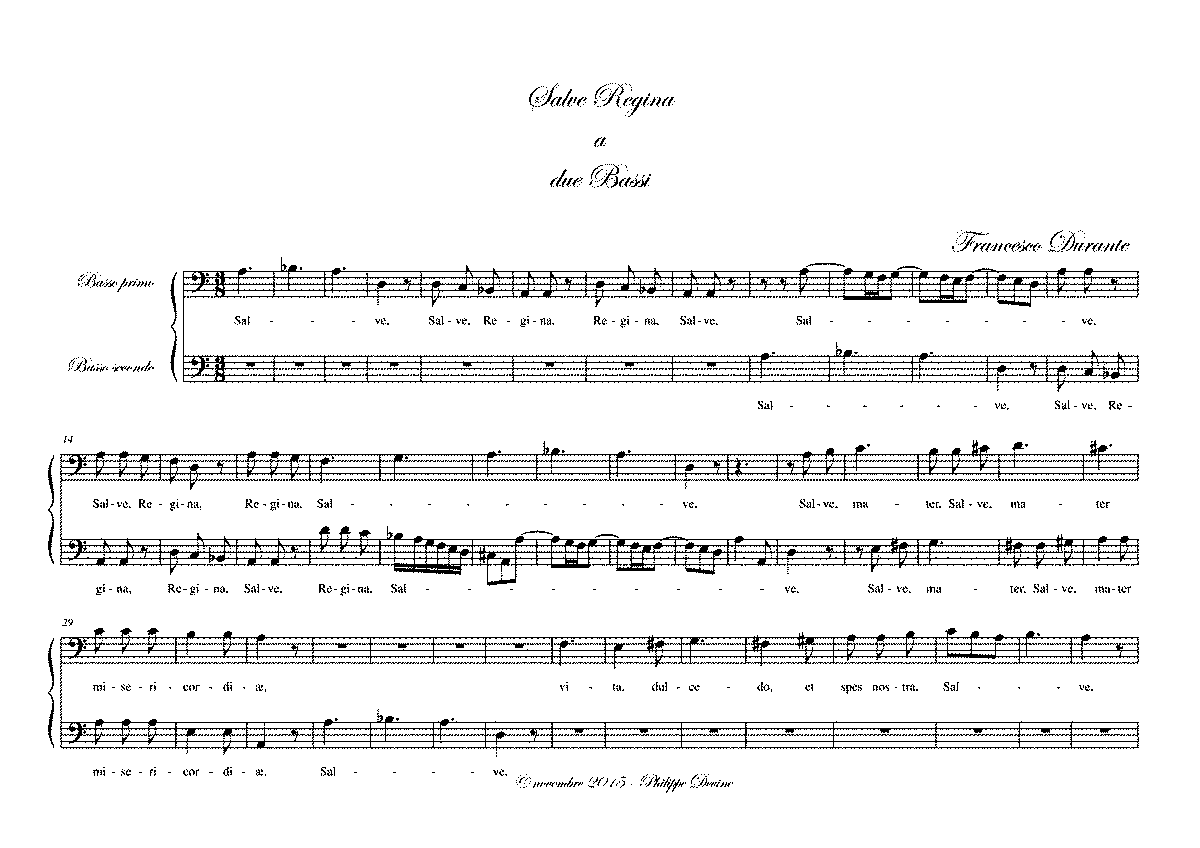

Supplement: S1 Dataset — We obtained the music score images from publicly available music databases, specifically from the IMSLP (International Music Score Library Project). This platform offers a vast collection of music scores that are in the public domain, as well as some modern works with explicit permission. The music score images can be accessed through the following link: https://imslp.org/ The majority of the music score images are sourced from the public domain, and therefore do not have any copyright restrictions. Specifically, the scores selected in our study belong to works in the public domain on the IMSLP platform, which are not protected by current copyright laws (ZIP) [file pone.0323447.s001.zip › PMLP1079251.png]

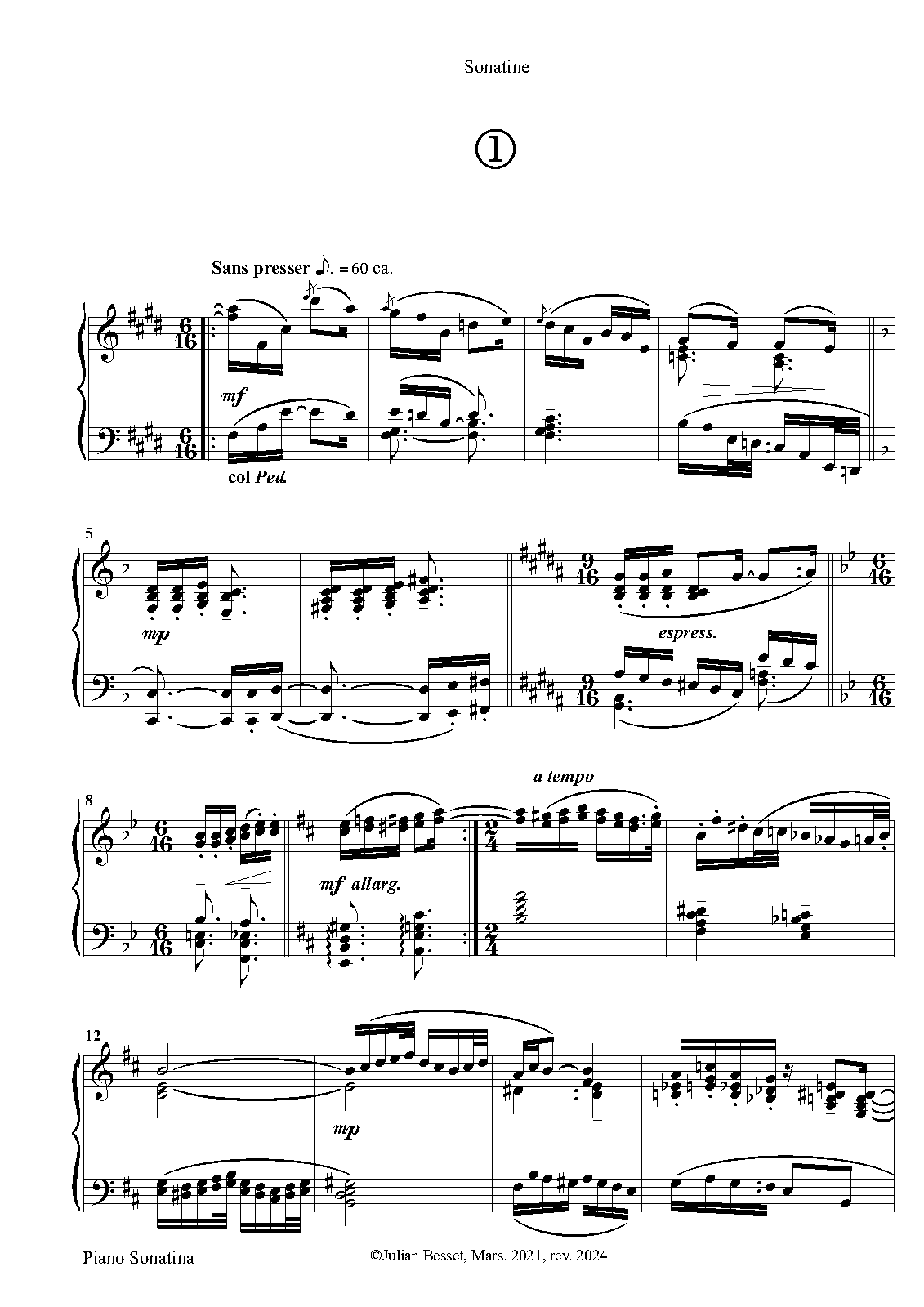

Supplement: S1 Dataset — We obtained the music score images from publicly available music databases, specifically from the IMSLP (International Music Score Library Project). This platform offers a vast collection of music scores that are in the public domain, as well as some modern works with explicit permission. The music score images can be accessed through the following link: https://imslp.org/ The majority of the music score images are sourced from the public domain, and therefore do not have any copyright restrictions. Specifically, the scores selected in our study belong to works in the public domain on the IMSLP platform, which are not protected by current copyright laws (ZIP) [file pone.0323447.s001.zip › PMLP1105014.png]

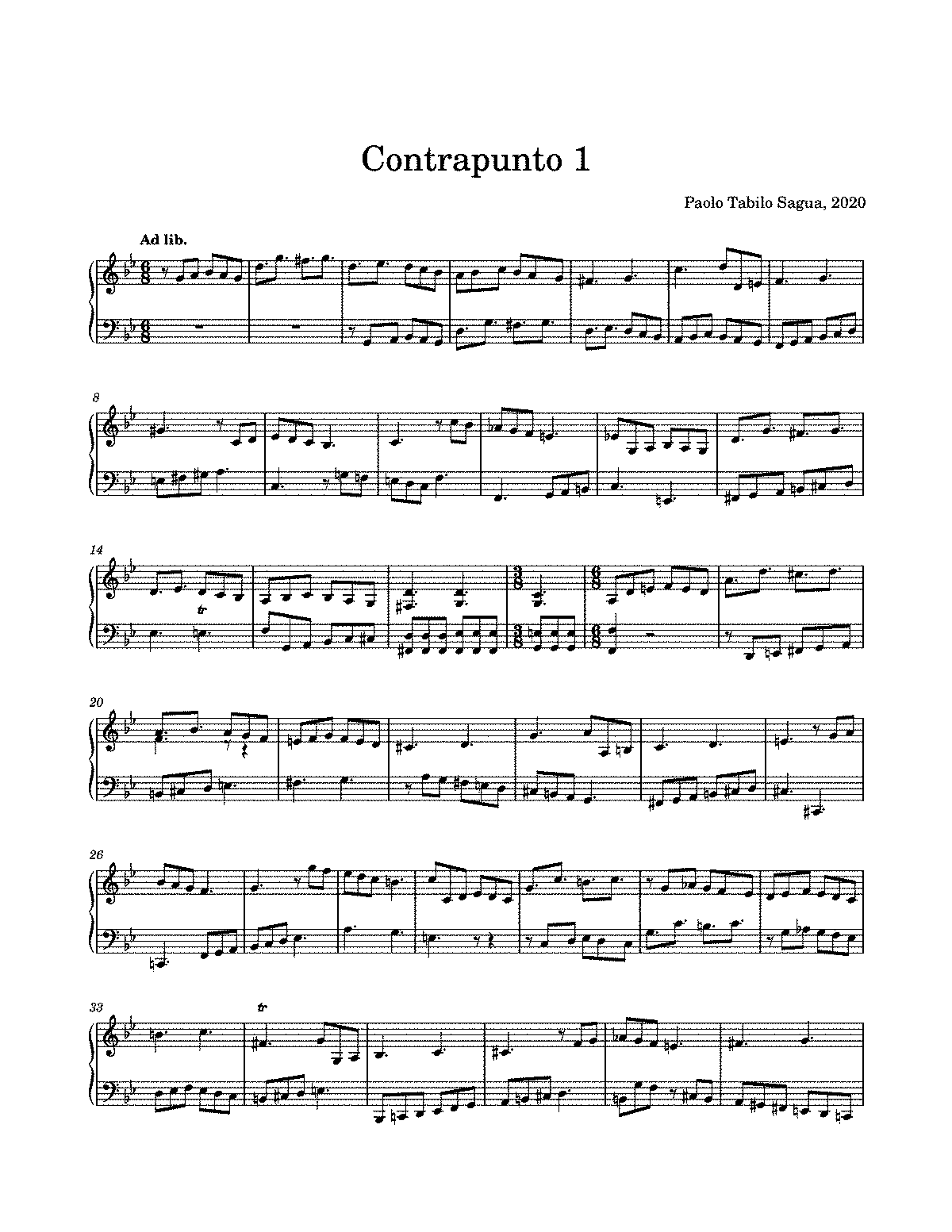

Supplement: S1 Dataset — We obtained the music score images from publicly available music databases, specifically from the IMSLP (International Music Score Library Project). This platform offers a vast collection of music scores that are in the public domain, as well as some modern works with explicit permission. The music score images can be accessed through the following link: https://imslp.org/ The majority of the music score images are sourced from the public domain, and therefore do not have any copyright restrictions. Specifically, the scores selected in our study belong to works in the public domain on the IMSLP platform, which are not protected by current copyright laws (ZIP) [file pone.0323447.s001.zip › PMLP1123946.png]

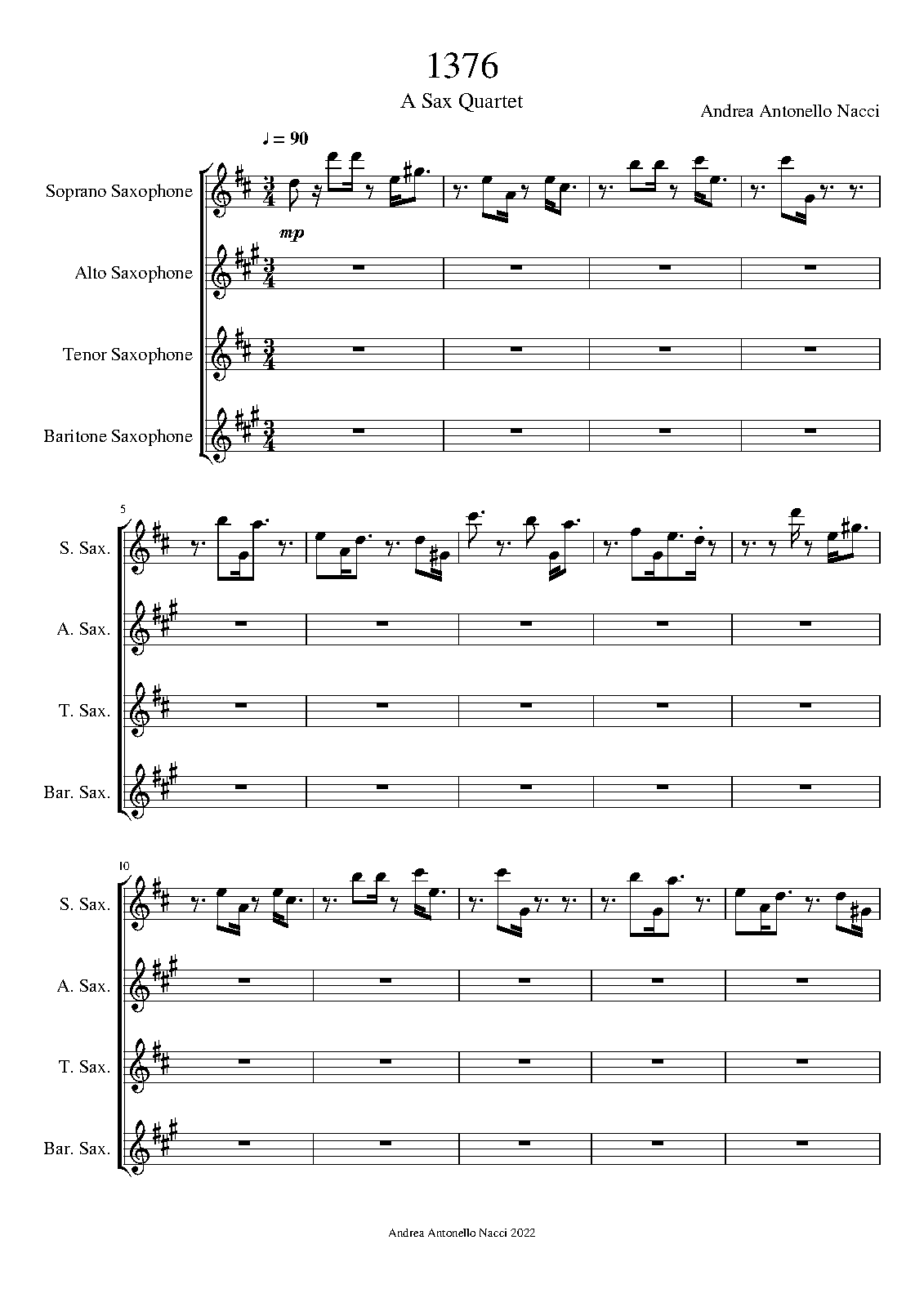

Supplement: S1 Dataset — We obtained the music score images from publicly available music databases, specifically from the IMSLP (International Music Score Library Project). This platform offers a vast collection of music scores that are in the public domain, as well as some modern works with explicit permission. The music score images can be accessed through the following link: https://imslp.org/ The majority of the music score images are sourced from the public domain, and therefore do not have any copyright restrictions. Specifically, the scores selected in our study belong to works in the public domain on the IMSLP platform, which are not protected by current copyright laws (ZIP) [file pone.0323447.s001.zip › PMLP1235492.png]

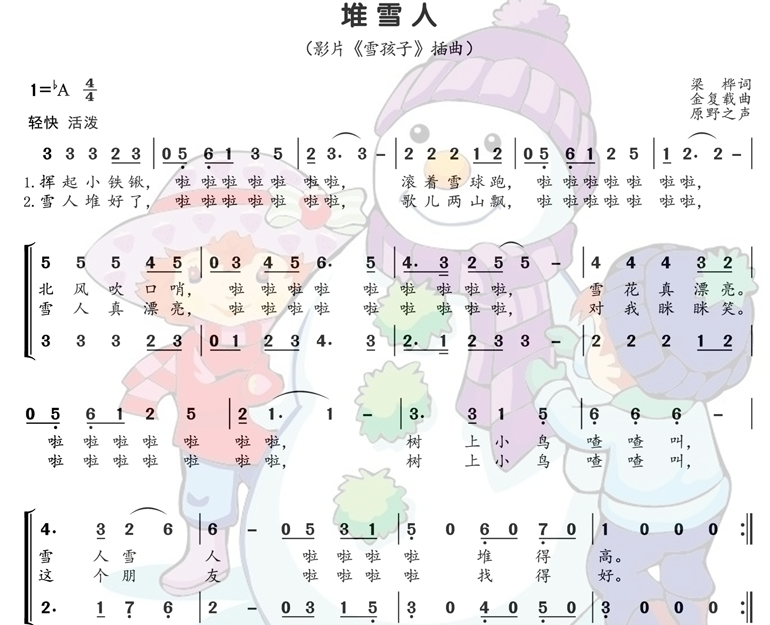

Supplement: S1 Dataset — We obtained the music score images from publicly available music databases, specifically from the IMSLP (International Music Score Library Project). This platform offers a vast collection of music scores that are in the public domain, as well as some modern works with explicit permission. The music score images can be accessed through the following link: https://imslp.org/ The majority of the music score images are sourced from the public domain, and therefore do not have any copyright restrictions. Specifically, the scores selected in our study belong to works in the public domain on the IMSLP platform, which are not protected by current copyright laws (ZIP) [file pone.0323447.s001.zip › R-C_remake.png]

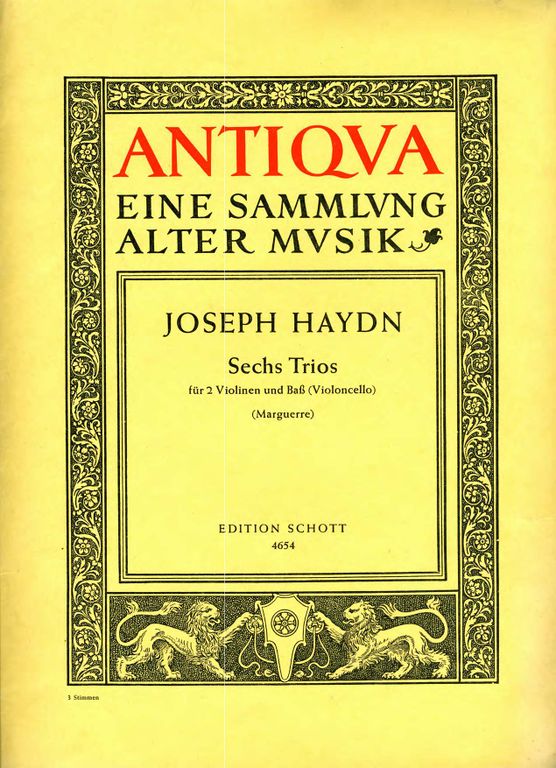

Supplement: S1 Dataset — We obtained the music score images from publicly available music databases, specifically from the IMSLP (International Music Score Library Project). This platform offers a vast collection of music scores that are in the public domain, as well as some modern works with explicit permission. The music score images can be accessed through the following link: https://imslp.org/ The majority of the music score images are sourced from the public domain, and therefore do not have any copyright restrictions. Specifically, the scores selected in our study belong to works in the public domain on the IMSLP platform, which are not protected by current copyright laws (ZIP) [file pone.0323447.s001.zip › String Trio in A major.png]

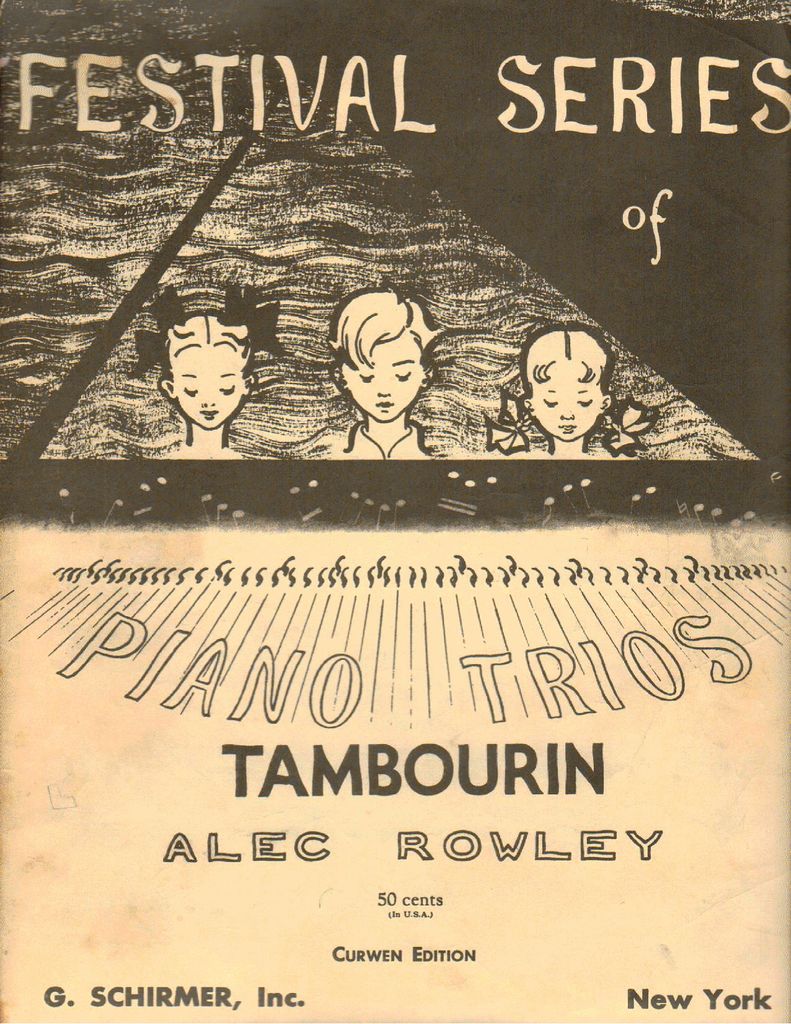

Supplement: S1 Dataset — We obtained the music score images from publicly available music databases, specifically from the IMSLP (International Music Score Library Project). This platform offers a vast collection of music scores that are in the public domain, as well as some modern works with explicit permission. The music score images can be accessed through the following link: https://imslp.org/ The majority of the music score images are sourced from the public domain, and therefore do not have any copyright restrictions. Specifically, the scores selected in our study belong to works in the public domain on the IMSLP platform, which are not protected by current copyright laws (ZIP) [file pone.0323447.s001.zip › Tambourin.png]

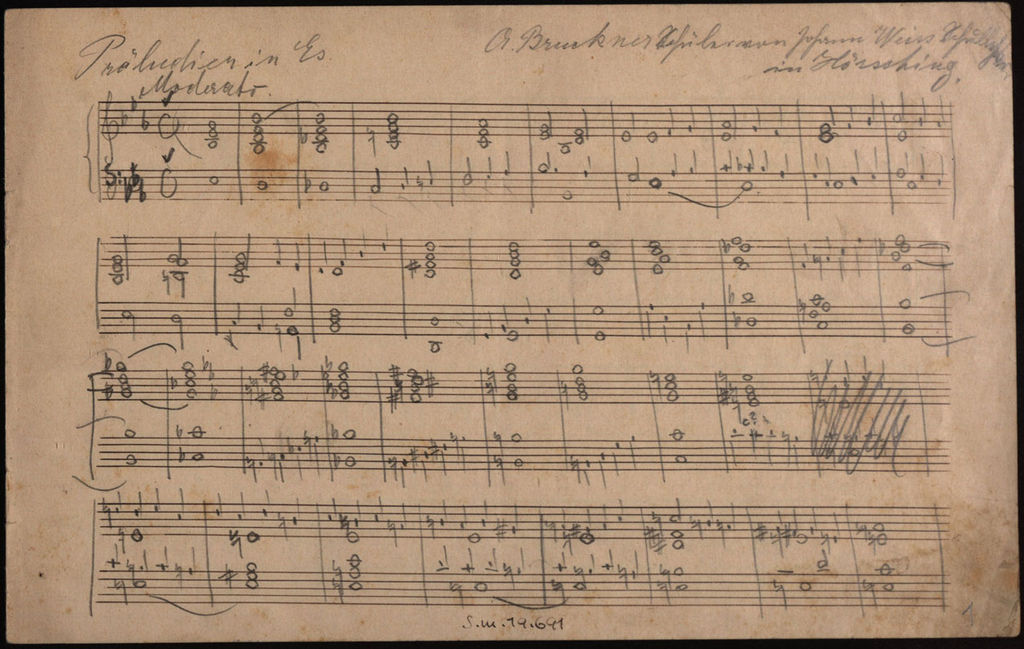

Supplement: S1 Dataset — We obtained the music score images from publicly available music databases, specifically from the IMSLP (International Music Score Library Project). This platform offers a vast collection of music scores that are in the public domain, as well as some modern works with explicit permission. The music score images can be accessed through the following link: https://imslp.org/ The majority of the music score images are sourced from the public domain, and therefore do not have any copyright restrictions. Specifically, the scores selected in our study belong to works in the public domain on the IMSLP platform, which are not protected by current copyright laws (ZIP) [file pone.0323447.s001.zip › TN-ABruckner.png]

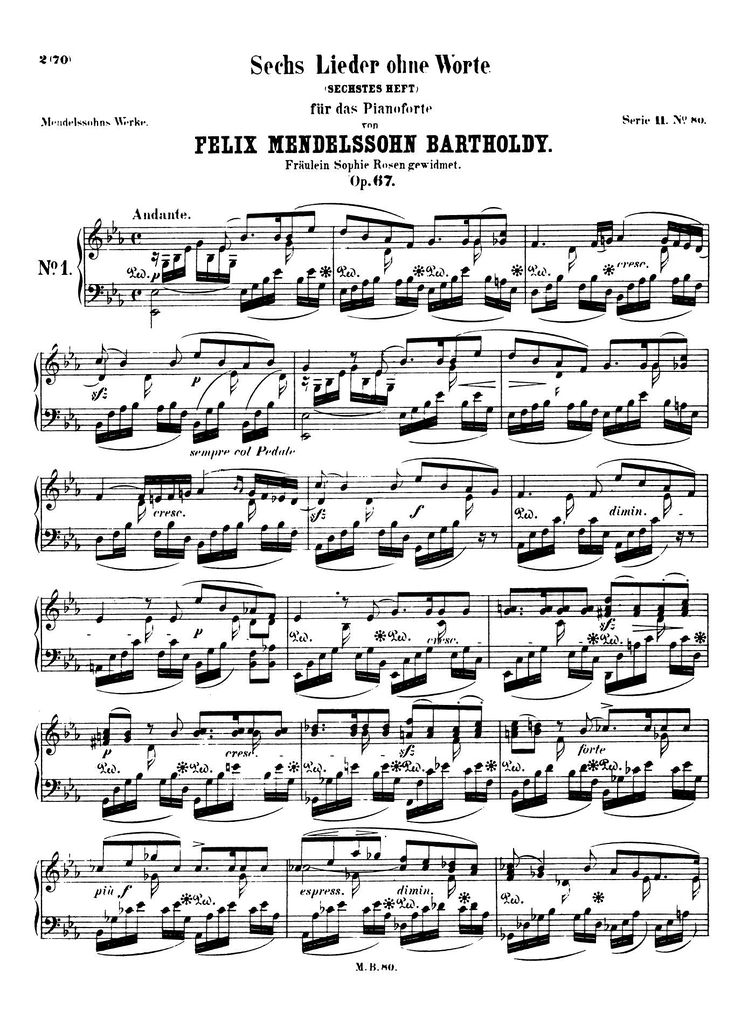

Supplement: S1 Dataset — We obtained the music score images from publicly available music databases, specifically from the IMSLP (International Music Score Library Project). This platform offers a vast collection of music scores that are in the public domain, as well as some modern works with explicit permission. The music score images can be accessed through the following link: https://imslp.org/ The majority of the music score images are sourced from the public domain, and therefore do not have any copyright restrictions. Specifically, the scores selected in our study belong to works in the public domain on the IMSLP platform, which are not protected by current copyright laws (ZIP) [file pone.0323447.s001.zip › TN-Mendelssohn.png]

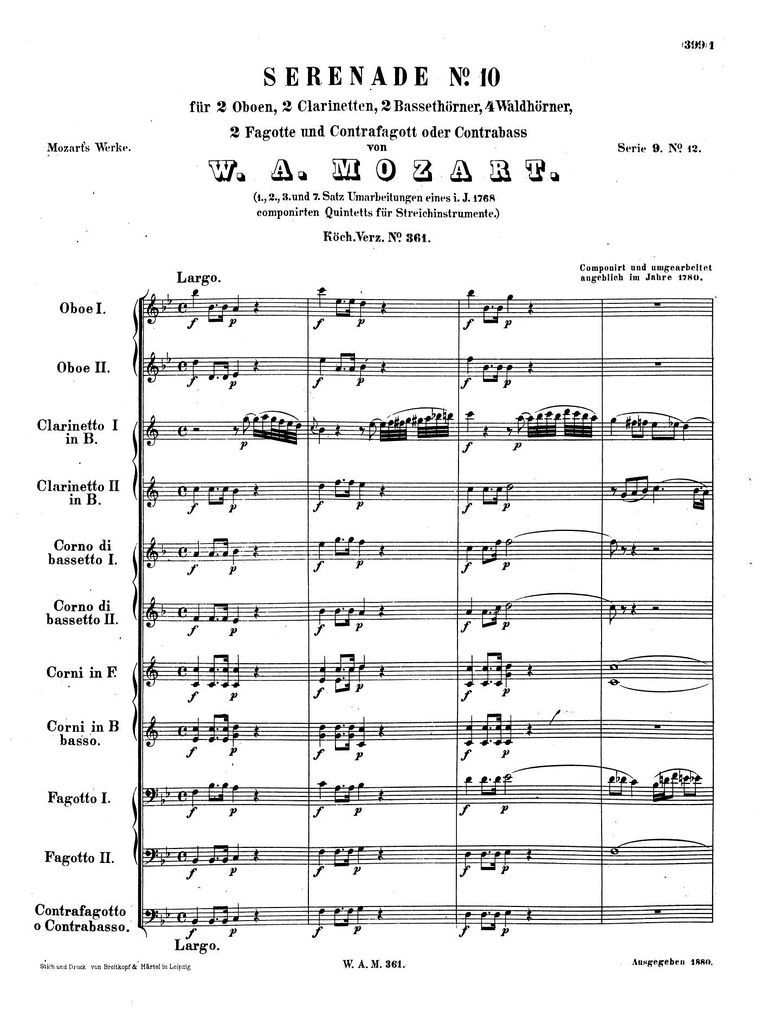

Supplement: S1 Dataset — We obtained the music score images from publicly available music databases, specifically from the IMSLP (International Music Score Library Project). This platform offers a vast collection of music scores that are in the public domain, as well as some modern works with explicit permission. The music score images can be accessed through the following link: https://imslp.org/ The majority of the music score images are sourced from the public domain, and therefore do not have any copyright restrictions. Specifically, the scores selected in our study belong to works in the public domain on the IMSLP platform, which are not protected by current copyright laws (ZIP) [file pone.0323447.s001.zip › TN-Mozart.png]

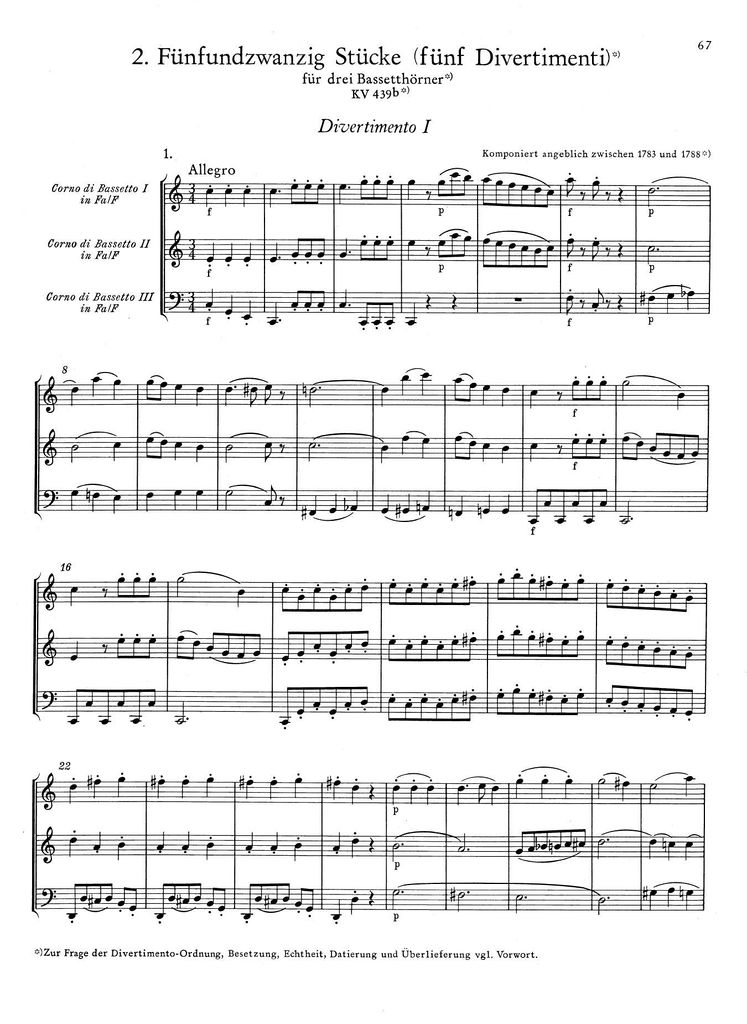

Supplement: S1 Dataset — We obtained the music score images from publicly available music databases, specifically from the IMSLP (International Music Score Library Project). This platform offers a vast collection of music scores that are in the public domain, as well as some modern works with explicit permission. The music score images can be accessed through the following link: https://imslp.org/ The majority of the music score images are sourced from the public domain, and therefore do not have any copyright restrictions. Specifically, the scores selected in our study belong to works in the public domain on the IMSLP platform, which are not protected by current copyright laws (ZIP) [file pone.0323447.s001.zip › TN-Mozart2.png]

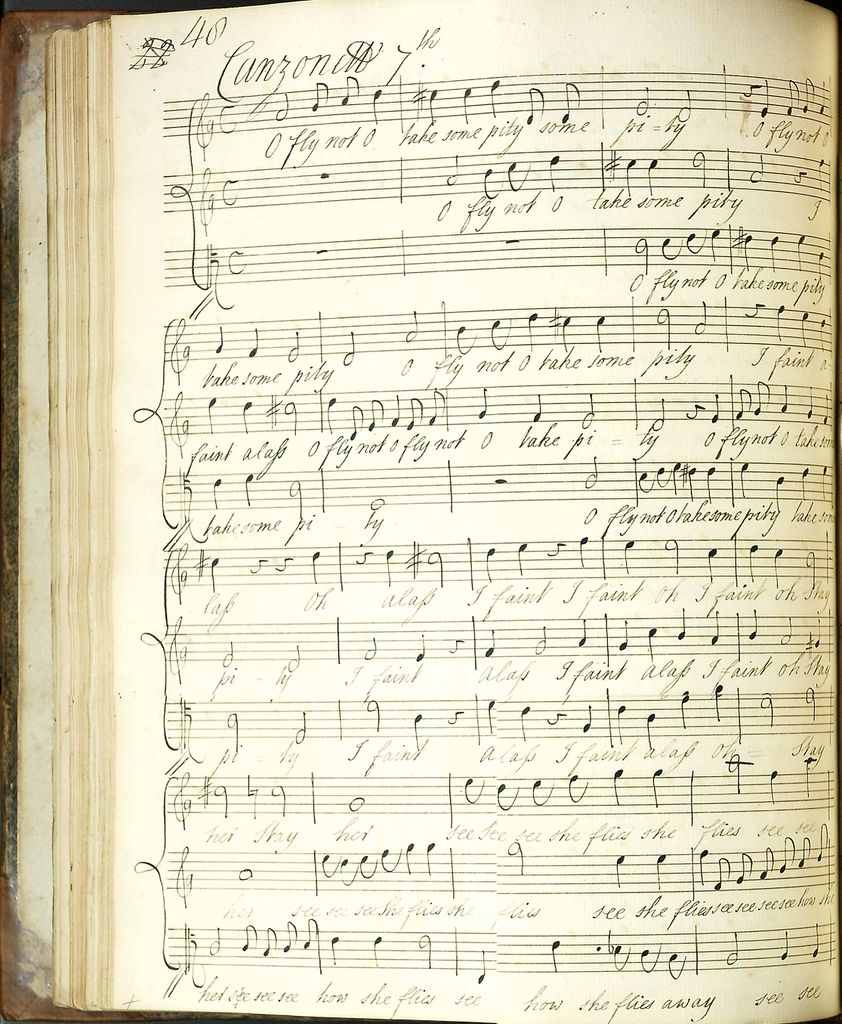

Supplement: S1 Dataset — We obtained the music score images from publicly available music databases, specifically from the IMSLP (International Music Score Library Project). This platform offers a vast collection of music scores that are in the public domain, as well as some modern works with explicit permission. The music score images can be accessed through the following link: https://imslp.org/ The majority of the music score images are sourced from the public domain, and therefore do not have any copyright restrictions. Specifically, the scores selected in our study belong to works in the public domain on the IMSLP platform, which are not protected by current copyright laws (ZIP) [file pone.0323447.s001.zip › TN-PMLP447851-MS.png]
