# Supplementary material for: Music score copyright protection based on mixed low-order quaternion Franklin moments
Source: PLoS One. 2025 Aug 25;20(8):e0323447. doi: 10.1371/journal.pone.0323447 (PMC12377592; doi:10.1371/journal.pone.0323447)
Supplement: Attachment — Submitted filename: Response_Letter.pdf [file pone.0323447.s002.pdf]

Dear Academic Editor Sadiq H. Abdulhussain:
No.: PONE-D-24-52606

We are very grateful to your and the reviewers' critical comments and thoughtful suggestions. Based on these comments and suggestions, we have made careful modification on the original manuscript. All changes made to the text are in red in the revised manuscript so that they may be easily identified. Some of your questions were answered below.

Once again, we acknowledge your comments and constructive suggestions very much, which are valuable in improving the quality of our manuscript.

Yours sincerely,
Qizheng Huang; JiaYi Zhu; Kang Huang; Yuanjie Xian; Jiyou Peng

Response

Here are our responses to the reviewers' comments one-by-one.

1 Response to reviewer 1

Comment 1.1

In general, the Abstract needs to specify the specific problem statements should be mentioned clearly to show the main contribution of this work. Besides, how the robustness has been increased using the new zero-watermarking method based on mixed low-order Franklin moments? In addition, based on the experimental results, how the proposed algorithm has strong resistance to mixed attacks? What measures have been used and what is the rate of improvement that has been achieved?

Response to 1.1:

Thanks very much for your comments. The revised abstract now explicitly states the core problem addressed by this work: (P1,Line1) Due to the rapid growth of the digital music industry, music copyrights have become valuable intangible assets for businesses, offering exclusivity and profitability. This article takes music copyrights as an example and designs a copyright protection method for music score digital images. The zero-watermarking algorithm offers an effective and lossless means of copyright protection. Owing to their geometric invariance, orthogonal moments exhibit superior robustness, positioning them as one of the mainstream methods in the research of zero-watermarking algorithms. The current zero-watermarking algorithms based on orthogonal moments face a trade-off between robustness and discriminability. In this paper, we propose a mixed low-order moments method based on quaternion-type fractional-order moments (QTFM), which balances the global information and texture details of color image contained in QTFM. Experimental results show that the mixed low-order moments method based on QTFM exhibits superior performance in terms of robustness. In the context of using mixed low-order moment features for image analysis, Franklin moments achieve higher average structural similarity (SSIM) values than other QTFMs.

In addition, we provide a more detailed discussion of the issues you raised in the discussion section following the experimental section of the paper.

(P18,Line324) The stability of the experimental results can be attributed to the following factors: (1) The mixed low-order moments method is used to extract low-frequency information from color images, making it is robust to image attacks that alter pixel values, such as noise, blurring, and cutting. (2) References [56, 57] embed watermarking image information into the original image by altering the moments amplitudes. This method makes the algorithm sensitive to variations in image pixel values. The method employed in reference [58] requires the computation of high-order moments when the embedded watermark image has a large size, which increases the time complexity and results in relatively poor robustness. (3) The QFFM demonstrates a smaller MSRE in image reconstruction experience, indicating that, at the same maximum order, the QFFM method retains more image texture information. This enhances its robustness against geometric transformations, including scaling, stretching, and compression. (4) Excessive image texture information will lead to increased sensitivity of the algorithm to local pixel changes. Therefore, combining QFFM with the mixed low-order moments method achieves a balance between global and local image information, enhancing the stability of the proposed algorithm against various types of image attacks.

(P18,Line320) When using the mixed low-order moment method, the average SSIM value increases around 7×10^{-5} compared to other types of orthogonal moments. Compared to references [55, 56, 57], the improvement is around 2.7×10^{-3}.

Comment 1.2

What is the difference between discrete and continuous moments in QTFM? Which one is preferable to use in color image processing? Why?

Response to 1.2:

Thank you for your question! In our revision, we have elaborated on the differences between discrete and continuous moments in QTFM and discussed their suitability for color image processing.

(P2,Line45) Discrete moments do not involve any approximation errors, making them more suitable for high-precision image processing tasks, such as image reconstruction, compression, and denoising.In contrast, polar continuous moments excel in rotation invariance, making them more suitable for applications requiring strong robustness.

Comment 1.3

The introduction section is weak and should be improved, more research should be added in this area. In addition, why does the ability of zero-watermarking algorithms based on orthogonal moments to resist geometric attacks remain limited? In addition, it is required to include some applications of orthogonal polynomials to show their importance such as: i) "A steganography based on orthogonal moments." Proceedings of the international conference on information and communication technology. 2019. ii) "Face recognition algorithm based on fast computation of orthogonal moments." Mathematics 10.15 (2022): 2721. iii) "3D object recognition using fast overlapped block processing technique." Sensors 22.23 (2022): 9209. Moreover, it is required to mention the main gaps that are handled in the proposed work. please list the main contributions of this work clearly.

Response to 1.3:

We apologize for the confusion caused by the imprecise wording in the original text. We have provided a more detailed explanation of the issue. Additionally, we have made comprehensive improvements to the introduction section.

(P2,Line24) Reference [13] proposes an image moment domain steganography algorithm based on orthogonal polynomials. ...

In response to your question, "In addition, why does the ability of zero-watermarking algorithms based on orthogonal moments to resist geometric attacks remain limited?" we have revised and elaborated on the original explanation to provide greater clarity and detail.

(P2,Line66) Through the analysis of the above content, the following conclusions can be drawn: (1) Zero-watermarking algorithms based on orthogonal moments have certain advantages over other methods in term of robustness. However, when watermark image has large sizes, some existing zero-watermarking algorithms require the computation of higher-order orthogonal moments to increase the capacity of the algorithm. In practical applications, high-order moments typically correspond to local features of the image, which means they exhibit lower robustness compared to low-order moments. (2) ...

(P2,Line53) The implicit construction of the classical Franklin function imposes specific constraints on its applicability. ...

Furthermore, we conduct a more comprehensive review of techniques related to orthogonal moments and added additional references [13, 14, 15, 16, 40, 41, 42, 43, 50, 52, 53, 54, 55, 56, 57].

Comment 1.4

It is recommended to add the section of the related works to provide a clear vision of the proposed work and its actual contribution in the context of other recent related papers.

Response to 1.4:

Thank you for your suggestion to improve the structure of the manuscript! As per your recommendation, we have added a section in the revised manuscript to provide a clearer vision of the research background and highlight the actual contributions of this work in the context of recent studies.

(P2,Line66) Through the analysis of the above content, the following conclusions can be drawn: ...

(P3,Line78) The main contributions of this paper can be summarized as follows:

- The paper employs non-recursive matrix operations to implement the orthogonalization process of Franklin polynomials, avoiding the high time complexity and computational errors associated with numerical integration by directly solving for the elements.
- Construction method of mixed low-order moments feature is used to arrange and quantize QTFM, effectively enhancing the robustness and discriminability of the zero-watermarking algorithm.
- The zero-watermarking algorithm constructed using quaternion Franklin moments achieves a balance between global and local image information, enhancing the algorithm's stability against various types of image attacks.

Comment 1.5

There are many grammatical and typo errors that should be checked in the entire manuscript.

Response to 1.5:

Thank you for your suggestion regarding the language quality! We have conducted a thorough grammar and typo check throughout the manuscript.

Comment 1.6

Please cite any specific figures, dataset, equation, and measurements (such as SSIM and PSNR) with reliable sources unless they are related to the authors.

Response to 1.6:

Thank you for your valuable suggestion! We have thoroughly reviewed the manuscript and added proper citations for figures, datasets, equations, and evaluation metrics (such as SSIM and PSNR) where necessary. The specific modifications are as follows:

(P11,Line251) The dataset used in this paper is composed of both computer-generated dataset [50] and music score images collected from the internet.

(P12,Line268) Using higher-order moments for image reconstruction is an effective method for assessing the accuracy of quaternion moments, where the reconstructed image can be evaluated using the mean squared reconstruction error (MSRE) [52]:...

(P14,Line287) Structural similarity (SSIM) [53] used to evaluate the robustness of algorithm is defined as: ...

(P14,Line292) The Peak Signal-to-Noise Ratio (PSNR) [54] can be used to assess the distortion level of the attacked image, which is defined as: ...

Comment 1.7

The mathematical formula of the time complexity of recursive and non-recursive orthogonalization process are given in section 2.2. However, it is not mentioned for the proposed one. Please clarify. Besides, it is preferable to put Fig 2 with the section of the Experimental results and discussions.

Response to 1.7:

Thank you for your valuable suggestions! We have made the following revisions to the manuscript as per your recommendations:

(P12,Line252) The time complexity of the non-recursive form in Eq.(4) is approximately $O(Dn^3)$, where D represents the grid density for numerical integration. Since all elements in the matrix correspond to the partition η_2, the time complexity of proposed method given in Eq.(5) is $O(3n^3)$.

We agree that placing Figure 2 in the "Experimental Results and Discussions" section would be more appropriate. Therefore, we have made this adjustment in the revised manuscript.

We place it in Section 5.1 Effectiveness of the Franklin Polynomial Calculation Method

Comment 1.8

How Gaussian and wavelet integration methods provide an accurate computation of GKS-QFFM. Please give more explanations.

Response to 1.8:

Thank you for raising this important question! Below, we provide further explanations on how Gaussian and wavelet integration methods enable accurate computation: We have added Section 5.2, which illustrates and validates the precise computation methods through image reconstruction experiments.

(P12,Line268) Using higher-order moments for image reconstruction is an effective method for assessing the accuracy of quaternion moments, where the reconstructed image can be evaluated using the mean squared reconstruction error (MSRE) [52]: ...

Comment 1.9

In general, the figures and results need more discussion with more explanation about each Tables.

Response to 1.9:

Thank you for your valuable suggestion! We have enhanced the descriptions and analyses of the figures and tables in the manuscript.

(P15,Line297) In this section, the robustness of the proposed algorithm is evaluated for the common geometric attacks, image processing attacks, and mixed image attacks. In the conducted experiments ...

(P18,Line320) When using the mixed low-order moment method, the average SSIM value increases around 7×10^{-5} compared to other types of orthogonal moments. Compared to references [55, 56, 57], the improvement is around 2.7×10^{-3}. The stability of the experimental results can be attributed to the following factors: ...

Comment 1.10

The conclusion section is good, but it needs more improvement. It should restart the problem statement, summarize the key findings, and demonstrate the significance of the current work.

Response to 1.10:

Thank you for your suggestion on improving the conclusion section! We have revised and enhanced this section based on your feedback. The specific changes are as follows:

(P19,Line348) A zero-watermarking algorithm based on mixed low-order Franklin moments is proposed for to address issue of copyright protection for digital music score images. The algorithm is constructed based on a set of fractional-order parameters, which are varied within a specific range. Subsequently, a quantization scheme is applied to the extracted features to generate a binary sequence for watermark generation and extraction. The algorithm combines the ability of mixed low-order moments method to effectively extract global image features with the capacity of Franklin moments to capture detailed local information in color images, thereby enhancing its robustness against various types of image attacks. Numerical experiments obviously show that our proposal is much more robust compared to the other considered methods and QTFMs. These results clearly demonstrate that our proposed method can be effectively against common image processing operations and mixed image attacks.

(P19,Line360) Although the proposed fast computation approach for Franklin functions has achieved certain success, numerical instability issues persist when dealing with high-order (order$>$ 200) Franklin polynomials. Therefore, developing a numerically stable construction of Franklin functions is a crucial direction for future work. Furthermore, the derivatives of classical Franklin functions demonstrate discontinuities at the boundaries of the segments. Consequently, the development of a smoother generalization of the Franklin functions represents a significant objective for future research.

2 Response to reviewer 2

Comment 2.1

The author use some incomplete senescence. For example, the author mentioned Experimental results demonstrate that the algorithm has strong resistance to mixed attacks. The author need to mentioned the which algorithm.

Response to 2.1:

Thank you for pointing out this issue! We have thoroughly reviewed the manuscript and made the following revisions:
(P1,Line1) Experimental results show that the mixed low-order moments method based on QTFM exhibits superior performance in terms of robustness. In the context of using mixed low-order moment features for image analysis, Franklin moments achieve higher average structural similarity (SSIM) values than other QTFMs.

Comment 2.2

The paper contributions need to be highlighted in the introduction. I would suggest using bullet points to make it clear to the reader.

Response to 2.2:

Thank you for your valuable suggestion! We have revised the introduction section to highlight the main contributions of this paper. The revisions are as follows:
(P3,Line78) The main contributions of this paper can be summarized as follows:

- The paper employs non-recursive matrix operations to implement the orthogonalization process of Franklin polynomials, avoiding the high time complexity and computational errors associated with numerical integration by directly solving for the elements.
- Construction method of mixed low-order moments feature is used to arrange and quantize QTFM, effectively enhancing the robustness and discriminability of the zero-watermarking algorithm.
- The zero-watermarking algorithm constructed using quaternion Franklin moments achieves a balance between global and local image information, enhancing the algorithm's stability against various types of image attacks.

Comment 2.3

Spacing should be exist between the text and reference. Please consider this point. For example ng[12].

Response to 2.3:

Thank you for your valuable feedback! We have thoroughly reviewed the manuscript, specifically addressing the issue of spacing between the text and references.

Comment 2.4

After eq 1 the author mentioned By Sn denotes. I would suggest using where Sn denotes.

Response to 2.4:

Thank you for your suggestion! We have revised the description following Eq. 1 as per your recommendation:
(P3,Line102) where S_n denote the space of functions defined on $[0, 1]$...

Comment 2.5

After each section, at the beginning of each section, the author needs to mention what this sections stands for. What are the contents of the section. It would be more clear to reader. Please address this issue.

Response to 2.5:

Thank you for your valuable suggestion! We have revised the manuscript to include a clear introduction at the beginning of each section, improving its structure and readability. The specific changes are as follows:

(P3,Line95) This section will introduce the definition of the classical Franklin function and presents a method for its fast and accurate computation. In addition, the quaternion type fractional-order moments(QTFM) is defined and explained.

(P5,Line145) In this section, the computational process of Franklin moments and necessity of precise calculation are presented. Accurate computation methods for Franklin moments are proposed using Gaussian integration and wavelet integration.

(P8,Line196) In this section, the construction method of mixed low-order moments feature and the processes of zero-watermarking algorithm are described.

(P11,Line249) In this section, a set of numerical experiments are conducted to evaluate the performance of the proposed zero-watermarking algorithm and compare it with that of the existing methods for real RGB images. The dataset used in this paper is composed of both computer-generated dataset [50] and music score images collected from the internet.

Comment 2.6

The authors should tell the reader about the computational overhead and latency introduced by the proposed method. Just clarification is needed.

Response to 2.6:

Thank you for your suggestion! We have added clarifications regarding the computational overhead and latency introduced by the proposed method. The revisions are as follows:

In fig 9, we evaluate the processing time of different QTFM methods based on Gaussian integration, wavelet integration, direct computation, and FFT. In fig 7, we analyze the time complexity associated with the calculation methods of Franklin polynomials.

(P12,Line261) The time complexity of the non-recursive form in Eq.(4) is approximately $O(Dn^3)$, where D represents the grid density for numerical integration. Since all elements in the matrix correspond to the partition η_2, the time complexity of proposed method given in Eq.(5) is $O(3n^3)$.

(P12,Line278) Fig 9 illustrates the comparison of time complexity between the direct computation method and the exact computation method. Since the Gaussian integration and wavelet integration methods directly increase the grid density of the discrete numerical integration, their time complexity increases significantly. Therefore, the direct computation method is used in the zero-watermarking algorithm to obtain the mixed low-order moments.

Comment 2.7

What are the limitations of the proposed approach. Could you please clarify this.

Response to 2.7:

Thank you for your insightful question! We have added a discussion on the limitations of the proposed approach before the conclusion section. The clarifications are as follows:

(P18,Line340) Although the proposed method has several advantages, it also has some limitations. Compared to other QTFM methods that also utilize mixed low-order moments, Franklin moments demonstrate superior average SSIM values in resisting various types of image attacks. However, for specific types of image attacks, it is possible to identify better QTFM methods, indicating that Franklin moments lack specificity in certain scenarios. Additionally, since Franklin functions are composed of piecewise linear functions, they exhibit higher time complexity as shown in Fig 9.

Comment 2.8

The author use "Where" after equation 4. It should be where small letter and no spacing at the beginning of the line.

Response to 2.8:

Thank you for your suggestion! We have replaced "Where" with "where" and conducted a comprehensive review of the manuscript to ensure that all descriptions following equations adhere to similar formatting standards.

Comment 2.9

Dot should be placed after fig 8 caption.

Response to 2.9:

Thank you for your detailed suggestion! We have made the corrections to the Fig 8 caption.

Comment 2.10

The author should state a future work and what can be carried out in the future. This would be really helpful and open the potential for future work.

Response to 2.10:

Thank you for your valuable suggestion! We have added a discussion on future work in the conclusion section to provide readers with potential directions for further research. The revisions include:

(P19,Line360) Although the proposed fast computation approach for Franklin functions has achieved certain success, numerical instability issues persist when dealing with high-order (order$>$ 200) Franklin polynomials. Therefore, developing a numerically stable construction of Franklin functions is a crucial direction for future work. Furthermore, the derivatives of classical Franklin functions demonstrate discontinuities at the boundaries of the segments. Consequently, the development of a smoother generalization of the Franklin functions represents a significant objective for future research.

Comment 2.11

Some relevant references are missing. For example [R1] and [R2] [R1] Mahmmod, Basheera M., Sadiq H. Abdulhussain, Tomáš Suk, Muntadher Alsabah, and Abir Hussain. "Accelerated and improved stabilization for high order moments of Racah polynomials." IEEE Access (2023).
[R2] Abdulhussain, Sadiq H., Basheera M. Mahmmod, Marwah Abdulrazzaq Naser, Muntadher Qasim Alsabah, Roslizah Ali, and S. A. R. Al-Haddad. "A robust handwritten numeral recognition using hybrid orthogonal polynomials and moments." Sensors 21, no. 6 (2021): 1999.

Response to 2.11:

Thank you for your valuable suggestion! We conduct a more comprehensive review of techniques related to orthogonal moments and added additional references [13, 14, 15, 16, 40, 41, 42, 43, 50, 52, 53, 54, 55, 56, 57].
